# Characterization of Biodegradable Food Contact Materials under Gamma-Radiation Treatment

**DOI:** 10.3390/ma16020859

**Published:** 2023-01-16

**Authors:** Karolina Wiszumirska, Dorota Czarnecka-Komorowska, Wojciech Kozak, Marta Biegańska, Patrycja Wojciechowska, Maciej Jarzębski, Katarzyna Pawlak-Lemańska

**Affiliations:** 1 Department of Industrial Products and Packaging Quality, Institute of Quality Science, Poznan University of Economics and Business, Al. Niepodległosci 10, 61-875 Poznan, Poland; 2 Polymer Processing Division, Institute of Materials Technology, Faculty of Mechanical Engineering, Poznan University of Technology, Piotrowo 3, 61-138 Poznan, Poland; 3 Department of Physics and Biophysics, Faculty of Food Science and Nutrition, Poznan University of Life Sciences, Wojska Polskiego 38/42, 60-637 Poznan, Poland; 4 Department of Technology and Instrumental Analysis, Institute of Quality Science, Poznan University of Economics and Business, Al. Niepodległości 10, 61-875 Poznan, Poland

**Keywords:** biodegradable polymer, packaging materials, gamma radiation, quality, safety, food contact materials

## Abstract

Radiation is an example of one of the techniques used for pasteurization and sterilization in various packaging systems. There is a high demand for the evaluation of the possible degradation of new composites, especially based on natural raw materials. The results of experimental research that evaluated the impact of radiation technology on biodegradable and compostable packaging materials up to 40 kGy have been presented. Two commercially available flexible composite films based on aliphatic–aromatic copolyesters (AA) were selected for the study, including one film with chitosan and starch (AA-CH-S) and the other with thermoplastic starch (AA-S). The materials were subjected to the influence of ionizing radiation from 10 to 40 kGy and then tests were carried out to check their usability as packaging material for the food industry. The results showed that the mechanical properties of AA-S films improved due to the radiation-induced cross-linking processes, while in the case of AA-CH-S films, a considerable decrease in the elongation at break was observed. The results also showed a decrease in the WVTR in the case of AA-S and no changes in barrier properties in the case of AA-CH-S. Both materials revealed no changes in the odor analyzed by sensory analysis. In the case of the AA-S films, the higher the radiation dose, the faster the biodegradation rate. In the case of the AA-CH-S film, the radiation did not affect biodegradation. The performed research enables the evaluation of the materials intended for direct contact with food. AA-CH-S was associated with unsatisfactory parameters (exceeding the overall migration limit and revealing color change during storage) while AA-S showed compliance at the level of tests carried out. The study showed that the AA-CH-S composite did not show a synergistic effect due to the presence of chitosan.

## 1. Introduction

The bioplastic market is developing an alternative to the conventional plastics used in the packaging industry [1,2,3,4]. Global bioplastics production capacities in 2021 reached 2.42 million tons. Currently, bioplastics constitute less than one percent of the more than 367 million tons of plastic produced annually [5,6]. However, with the increase in demand and the emergence of increasingly sophisticated biopolymers, applications, and products, this is a constantly growing market [7]. According to the latest market data developed by European Bioplastics, in cooperation with the nova-Institute research institute, the global production capacity of bioplastics will increase from around 2.11 million tons in 2018 to around 5.22 million tons in 2023 [5,6].

Bioplastics that are biobased, biodegradable, or both have similar, or even the same, properties as conventional plastics and offer additional environmental benefits, such as a reduced carbon footprint or additional waste management options, such as composting [8,9]. With the presence and availability of the bioplastics market, modified and improved properties, such as flexibility, durability, printability, transparency, barrier properties, heat resistance, gloss, and many more, have been significantly enhanced. Owing to these changes, bioplastics are becoming increasingly attractive to the demanding packaging industry [10,11,12,13,14].

Bioplastics are a large family of materials that can be divided into the following three main groups [15,16,17]: (i) biobased or partially biobased non-biodegradable plastics, such as biobased PE (polyethylene), PP (polypropylene), or PET (polyethylene terephthalate) (so-called drop-ins) and biobased technical performance polymers, such as PTT (polytrimethylene terephthalate); (ii) plastics that are both biobased and biodegradable, such as PLA (polylactide) and PHA (polyhydroxyalkanoates) or PBS (polybutylene succinate); (iii) plastics that are based on fossil resources and are biodegradable, such as PBAT (poly(butylene adipate-co-terephthalate) and PCL (polycaprolactone). Biobased or partially biobased durable plastics, such as biobased PE and PET, have the same properties as polymers produced from petroleum, this is why can be mechanically recycled in the existing recycling streams. In addition, materials such as PLA and PHA (produced from raw materials such as starch) have good barrier properties, which are important for the packaging industry, and are biodegradable and compostable (if confirmed by the appropriate certificate) [5,6,18,19,20].

Biodegradable shopping and waste collection bags are available on the consumer market. A new market potential (niche) is emerging for rigid bioplastics, including food packaging and take-out meals, disposable cutlery, and stationery. The portfolio of biodegradable products for the automotive and agriculture sectors is also expanding to the consumer electronics [21,22,23]. 

The components that appear in the tested materials in the context of the impact of gamma radiation are briefly discussed below. Aliphatic–aromatic copolyesters (AAC) are a hybrid combination characterized by good strength properties and resistance to the thermal degradation of aromatic polyesters and biodegradability, due to the presence of aliphatic polyesters [24,25]. Aromatic polyesters exhibit water hydrolytic resistance due to the hydrophobic benzene rings in their chemical structure, while aliphatic polyesters undergo spontaneous hydrolytic degradation under the influence of moisture because they contain short methylene chains separated by ester bonds. They are also biodegradable in the presence of enzymes [26]. AAC degradation carried out in compost under elevated temperature conditions is significantly different from degradation in aquatic environments. In liquid media, the AAC degradation process is usually much slower, which is mainly due to the lower temperature and composition of the microflora [27]. The aliphatic–aromatic polyesters include PBTA copolyesters (based on terephthalic acid, adipic acid, and 1,4-butanediol) and PBTS copolyesters (based on terephthalic acid, succinic acid, and 1,4-butanediol). The relatively low price of AAC compared to PHA and PCL, as well as its good utility and processing properties, favor the development of this group of plastics. Based on the efficiency tests of the radiolytic emission of hydrogen G(H_2_) during irradiation, it can be assumed that AAC is radiation-resistant in the scope of low-dose applications [28].

Starch is a polysaccharide that consists of amylose and amylopectin. The ratio of these polysaccharides depends on the source of starch (potato, rice, corn, etc.). The effect of ionizing radiation on starch is demonstrated through the ways in which glycosidic bonds break in the polymer and modification of its crystalline structure [29]. Materials based on starch are brittle and hydrophilic, which limits their processing and use. To overcome this problem, starch is mixed with various synthetic and natural polymers. Mixtures of starch with various compostable polymeric materials show insolubility or exhibit enhanced strength and other advantageous features. Usually, starch-based mixtures exhibit favorable strength, processing, and performance characteristics, e.g., they have greater water resistance [30,31].

Chitosan (poly [β-(1,4)-2-ammonium-2-deoxy-D-glucopyranose) is a pseudo-natural, non-toxic biopolymer [32,33,34]. It is made of N-acetyl-D-glucosamine and D-glucosamine residues connected by β-1,4-glycosidic bonds. It is industrially obtained by the chemical N-deacetylation of chitin, which is the main component of the cell wall of fungi of the class *Zygomycetes*, e.g., *Absidia, Mucor* and *Rhizopus*. It is also found in the outer skeletal structures of numerous invertebrates, including crustaceans and insects [35]. Its hydrophilic nature and sensitivity to changes in the pH of the environment mean that the stability of chitosan is much worse than that of chitin. It dissolves well in aqueous acid solutions. Crosslinking causes an increase in the space between the chains, which results in the partial degradation of its crystalline structure and a decrease in its solubility. Chitosan is a bioactive, biocompatible, biodegradable, non-toxic polymer with high adhesion, which is why it is used in many fields, including agriculture, environmental protection, food, and the cosmetics industry, as well in biomedicine [36,37,38,39]. Due to its antimicrobial properties, chitosan can be used in food products to extend their shelf life and as an ingredient in packaging films. Chitosan and its derivatives have strong biocidal activity against various groups of G(+) and G(−) bacteria, fungi, and viruses. The properties of chitosan depend on its molecular weight, degree of deacetylation, concentration, pH, and the composition of the environment in which it is located. Chitosan can be made into films that exhibit high gas barriers. However, their fragility requires the use of plasticizers such as polyols (glycerine, sorbitol and polyethylene glycol) and fatty acids (stearic and palmitic acid) [40]. Mathew and Abraham reported that the physical modification of starch–chitosan mixtures with gamma radiation or ultrasound can modify them through cross-linking, thereby improving the functionality of the materials [41]. 

When analyzing the possibility of using packaging materials in radiation technologies, the risk concerning the potential packaging–product interaction should be considered [42,43]. Gamma irradiation is not a surface treatment, because the photon energy is high enough to penetrate the materials [44]. The impact of ionizing radiation on the polymer may lead to the formation of free radicals, and the formation of reactive intermediates. These reactions may occur during direct contact and may be deferred in time and their effects may be delayed [45,46]. There is a risk leading to unsatisfactory barrier properties, or exceeding the limit of overall and specific migration, as stipulated by Regulation (EC) No. 1935/2004 of the European Parliament [47], Commission Regulation (EU) No. 10/2011 [48] and the requirements of the Commission Regulation (EC) No. 2023/2006 [49] for materials and articles intended to come into contact with food. 

Ionizing radiation is an alternative method to conventional methods of product preservation and sterilization, guaranteeing a high degree of hygienization during storage and use. Various food and industrial products can be subjected to radiation sterilization, for example surgical implants, medical utensils, special purpose food, or herbs and spices. The originality of the use of ionizing radiation to combat pathogens involves the sterilization of the packaging before filling or sterilization of the packaged product. Maintaining the specific properties of the product in the irradiation process and protection against secondary contamination requires the use of appropriate packaging, which must additionally meet the requirements of radiation treatment.

Research on the impact of ionizing radiation on packaging made of various materials, including plastic, has been carried out for many years, but there are few reports on biodegradable materials [50,51]. A prerequisite for considering the application potential of new solutions in radiation technologies is the identification of the possible changes caused by ionizing radiation. This is important because it can lead to the process of cross-linking or degradation of the material, and the chemical substances formed as a result of radiolysis can cause negative phenomena (e.g., sensory changes), which may only become apparent after a certain period.

The widespread use of biodegradable materials, as well as other packaging materials, requires verification of the compliance with the requirements in at least two aspects, which are as follows: the technological conditions in radiation pasteurization/sterilization and safety of use, which is reflected in the applicable law. The aim of the research was to determine whether the increasing dose of ionizing radiation up to 40 kGy affects the structure and properties of biodegradable films and flexible films for food packaging, including an aliphatic–aromatic copolyester with thermoplastic starch (AA-S) and an aliphatic–aromatic copolyester with chitosan and thermoplastic starch (AA-CH-S), and whether it is advisable to conduct further research with these materials and their composites. 

## 2. Materials and Methods

### 2.1. Materials

Tests were conducted on biodegradable and compostable flexible films for food packaging, including an aliphatic–aromatic copolyester with thermoplastic starch (AA-S) and an aliphatic–aromatic copolyester with chitosan and thermoplastic starch (AA-CH-S) as presented in Table 1. Both materials are commercially available and were purchased directly from the manufacturers. 

### 2.2. Mechanical Properties

The tensile properties of the films in the machine direction (MD) were tested according to the standard ISO 527-1 and ISO 523-3 [52,53], using a universal testing machine Zwick/Roell Z005 (Ulm, Germany) equipped with testXpert II V3.7 software. The tensile characteristics were measured at room temperature with a crosshead speed of 200 mm/min. Film tensile strength (σ_M_) and elongation at break (ε_B_) were evaluated. The reported data represent the average of 10 measurements. The samples before testing were climatized at 23 ± 2 °C, with an air humidity of 50 ± 5%. 

### 2.3. Overall Migration

Migration tests were performed based on the appropriate relative standards, which specify the type of contact between the packaging material and a simulant, and the time and temperature in which the test is carried out. The conditions of the test reflect risks that are higher than the real risks, while the principle of the “worst case scenario” ensures that the application of food contact materials (FCMs) is safe in their real conditions of use. Under EU/10/2011, all packaging materials should (which is understood as “must”) meet the requirements concerning overall migration (OML, overall migration limit) and specific migration (SML, specific migration limit). The OML means the total of the non-volatile substances that are transferred from the packaging material or article into a simulant (a liquid imitating food). The residue is expressed in milligrams and square decimetres (mg/dm^2^) on the sample’s surface, which is in direct contact with the simulant. OML cannot exceed 10 mg/dm^2^ (or 60 mg/kg of food stuff in the case of products intended for infants and young children) of the non-volatile substances that diffuse from the surface of the material, which comes into direct contact with food. In the case of packaging that is suitable for all kinds of foodstuff and food simulants, A (or water), B (acetic acid 3% (*w*/*v*)), and D2 (vegetable oil) are used for the migration test. The selection of test conditions and the methodology were decided based on the standard EN 1186-1, EN 1186-3 [54,55]. The method of total immersion was used, using two food simulants, water and food simulant D1 (ethanol 50% (*v*/*v*)), for 10 days at a temperature 60 °C. The contact surface of the material with the model fluid was 2.0 dm^2^, according to the guidelines of the above-mentioned standards. The film edge surface was omitted. The test was performed in five replicates for each of the model fluids (water and ethyl alcohol). 

### 2.4. Sensory Analysis of Packaging Materials

Organoleptic (olfactometric) evaluation is a test that is complementary to migration tests. It is a non-instrumental analysis carried out with the help of the human senses (sight, taste, and smell). The test allows the verification of whether the material releases volatile compounds that cause odor formation in the packaging material. Fragrances can arise from the degradation products of plastics or processing additives (dyes and plasticizers), solvents or printing inks. Sensory analysis can be extended with instrumental identification methods. Confirming the sensory indifference of the packaging material in direct contact with food is a key aspect of the packaging material’s compliance with EC/1935/2004 (Art. 3) [47]. Sensory evaluation was carried out following the standardized methods described in the standards. The test was conducted by an experienced team of six specialists in the field of sensory analysis. The samples were prepared based on the methodology of Regulation 10/2011 from 14 January 2011 on plastic materials and products intended to come into contact with food, which specifies the requirements and tests of global migration. Distilled water was chosen as the model fluid. The test was carried out following the methodology of DIN 10955: 2004 [56]. A five-grade scale of flavor or aroma intensity was used, where 0 means that there is no noticeable aroma transfer and no flavors; 1—the transfer of smell/unpleasant aftertaste is palpable (still difficult to define); 2—moderate odor/unpleasant aftertaste transfer (the assessor can generally determine the origin of the smell, but no specific substance can be identified); 3—moderately strong odor/unpleasant aftertaste; 4—strong transfer of smell/unpleasant aftertaste (the assessor can identify the responsible substance) [56]. It was assumed that the median of 3 or more results is inconsistent with the provisions of EC/1935/2004, art. 3 [47].

### 2.5. Oxygen Transmission Rate

The oxygen transmission rate (OTR) was determined by ASTM F3136 [57], using the OxyPerm system that consists of the Oxysense 325 oxygen analyzer and dedicated test chambers for oxygen permeation measurements. Periodic measurement of oxygen concentration within the test chambers during the permeation process was based on the fluorescence quenching time measurements obtained by ASTM F2714-08 [58]. 

The test samples were cut in the shape of squares with a side of 6.5 cm. Then, the samples were placed in the test chambers, which were flushed with nitrogen (5.0 purity) to obtain an oxygen-free atmosphere. Then, the OTR measurements began, consisting of the periodic measurement of the oxygen concentration inside the measuring chambers. It was assumed that the end of the study was the moment in which the correlation coefficient between the individual measurements was greater than 0.95. The OTR measurements (cc/m^2^/24 h) were performed in controlled conditions at a temperature of 23 ± 2 °C and a relative humidity of 60 ± 10%, respectively. The test was performed in three replicates for all variants of the investigated materials.

### 2.6. Water Vapor Transmission Rate

The water vapor transmission rate (WVTR) was determined according to ASTM E96/E96M-16 [59], using the desiccant method and the EZ-Cup Vapometer (Thwing-Albert Instrument Company, West Berlin, NJ, USA). The Vapometer consists of an aluminium cup and threaded flanged ring with two neoprene gaskets and with an additional Teflon seal that holds the specimen in place. Specimens were cut into a circular shape with a diameter of 74 mm. The measuring cups were first filled up to ¾ of their internal height with silica gel (previously dried), which was used as a water absorption agent (desiccant). The specimens were then placed between the two mentioned rubber gaskets, fitted onto the cup collar and pressed down using the threaded aluminium ring. The effective diameter of the specimen exposed to the environment was 63.5 mm. Next, the cups were placed in a controlled climatic test chamber that operated at a temperature of 23 ± 2 °C and a relative humidity of 60 ± 10%, respectively. The cups were weighed periodically using a laboratory balance until 10 data points were collected. The water-vapor transmission rate (*WVTR*) was calculated as follows [60]:

(1) $$WVTR=\frac{\Delta W}{t\cdot A}$$

where *WVTR* represents the water vapor transmission rate (g·m^−2^·d^−1^); Δ*W* is the weight change (g); *t* indicates the time of the experiment (h); *A* is the test area (m^2^).

### 2.7. Color Measurements

The color of the AA-CH-S and AA-S films was analyzed by using the tristimulus colorimeter MINOLTA CR310. Standard CIE conditions with illumination were used. The configuration included the illuminant D65 and an angle of 10. The readings were taken using the CIELAB system (L*, a*, b*), and presented as the L* value (color brightness). The results are expressed as the mean value of five measurements. Color was evaluated for the bioplastics AA-CH-S and AA-S before and after ionization irradiation after the 12-month storage period. Based on the obtained results, the total color difference (Δ*E*) of the irradiated film samples stored for 12 months concerning the non-irradiated material (sample 0) was calculated. Color change (Δ*E*) can be calculated from the following equation:

(2) $$\Delta E=\sqrt{{\left(\Delta L\right)}^{2}+{\left(\Delta a\right)}^{2}+{\left(\Delta b\right)}^{2}}$$

where *L* is the lightness (also referred to as luminance) parameter (maximum value of 100 represents a perfectly reflecting diffuser; the minimum value of zero represents the color black); *a* is the axis of the red–green character (*+a* = redder; *−a* = greener); *b* is the axis of the yellow–blue character (*+b* = yellower; *−b* = bluer) [61].

For the interpretation of data, the criterion of the International Commission on Illumination CIE regarding the acceptability of color was adopted. When 0 < Δ*E* < 1, the observer does not notice the color difference; when 1 < Δ*E* < 2, only an experienced observer notices the color difference; when 2 < Δ*E* < 3.5, the color difference is noticeable for an inexperienced observer; when 3.5 < Δ*E* < 5, the color difference is noticeable; when 5 < Δ*E*, the observer notices two different colors. 

### 2.8. Structure Analysis by Fourier Transform Infrared (FTIR) Spectroscopy

Spectral profiles of the non-irradiated and irradiated biofilms were recorded in the reflection mode in the range 4000–400 cm^−1^ with a resolution of 1 cm^−1^ by an attenuated total reflection Fourier transform infrared spectroscope (Perkin-Elmer Spectrum 100 IR, ATR, Waltham, MA, USA). Spectra of the biofilms were recorded at room temperature directly on the diamond crystal. Each spectrum recorded was an average of 16 successive scans. In addition, ten acquisitions were performed for each experiment by the manual rotation of biofilm samples. The ten replicates for each sample of non-irradiated and irradiated biofilms were averaged before further data processing and the mean spectra for each were used in subsequent analysis. Principal component analysis (PCA) was performed on the IR transmission spectra in the range of 4000–400 cm^−1^. The cross-validation method was used to check the models. Multivariate data analysis was performed using the Unscrambler 9.7 software (CAMO, Oslo, Norway).

### 2.9. Surface Morphology Using SEM Microscopy

The surface microstructure of the films was studied using a scanning electron microscope (SEM; Zeiss EVO 40 with the electron accelerating voltage of 17 kV). Before the analysis, the surfaces of the films were coated with gold (Sputter Coater SCD 050). The images were taken at the magnification of 500×.

### 2.10. Biodegradation in the Activated Sludge Environment

The biodegradation test was carried out under active sludge conditions. The test proceeded for 70 days at a temperature of 55 °C. Periodic weight loss measurements were applied using the weight method with an accuracy of 0.0001 g, every 14 days. Due to the equipment capabilities and long analysis time, samples irradiated under air conditions were used with two doses of 10 and 40 kGy; all the samples were run in triplicate. The presented results are a preliminary attempt to determine the biodegradability of the tested materials in a liquid environment.

## 3. Results and Discussion

### 3.1. Materials

The radiation process was conducted on a laboratory scale using a cobalt (^60^Co) gamma radiation chamber GC 5000 with a dose rate of 8.5 kGy/h. The following radiation doses were used: 10, 20, and 40 kGy. The samples were conditioned before radiation process under the following standardized conditions: 22 ± 2 °C and a relative air humidity of 65 ± 2%.

### 3.2. Mechanical Properties

**Figure 1:**
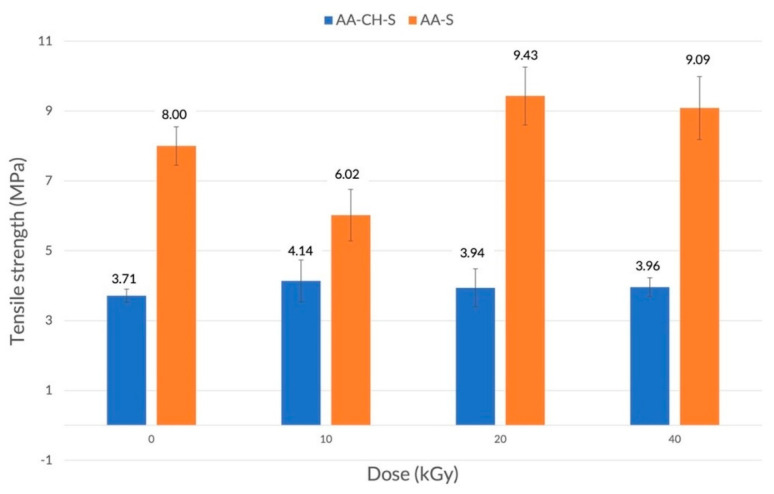
Tensile strength of AA-CH-S (blue) and AA-S (orange) films irradiated with doses of 10, 20, and 40 kGy in the machine direction.

**Figure 2:**
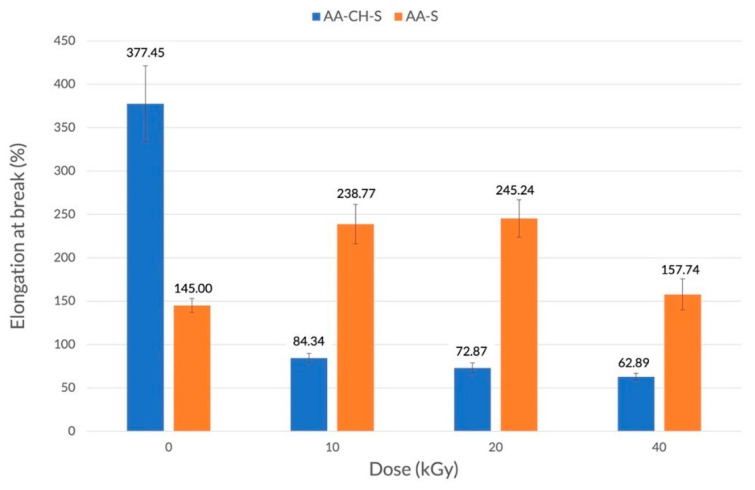
Elongation at break for AA-CH-S (blue) and AA-S (orange) films irradiated with doses of 10, 20, and 40 kGy in the machine direction.

In the case of the AA-CH-S film that contained three components (an aliphatic–aromatic copolymer, starch, and chitosan), the changes in the tensile strength caused by the radiation were small and rather accidental (Figure 1). A further increase in the radiation dose (from 10 to 40 kGy) did not cause a significant change. On the other hand, a significant decrease in the elongation at break was observed (Figure 2) from about 400% for the control sample to 50–80% in the case of the irradiated samples. This is possibly due to the phenomenon of creating additional bonds, and thus reducing chain mobility. It is noteworthy that the impact on the elongation at break values remained at a low level regardless, of the radiation dose applied (10, 20, or 40 kGy).

The AA-S film was characterized by higher mechanical strength than the AA-CH-S film (Figure 1). Interestingly, an increase in the radiation dose (from 10 to 20 and 40 kGy) caused a slight increase in the tensile strength values from 8 MPa for the control sample to 9.4 and 9.1 MPa, respectively. An increase in the elongation at break values, from 145% for the reference sample to 239% and 245% for the samples treated with the radiation doses of 10 and 20 kGy, respectively, was also observed (Figure 2). A further increase in the radiation dose to 40 kGy resulted in a decrease (157%) in the elongation at break value. 

### 3.3. Overall Migration

**Figure 3:**
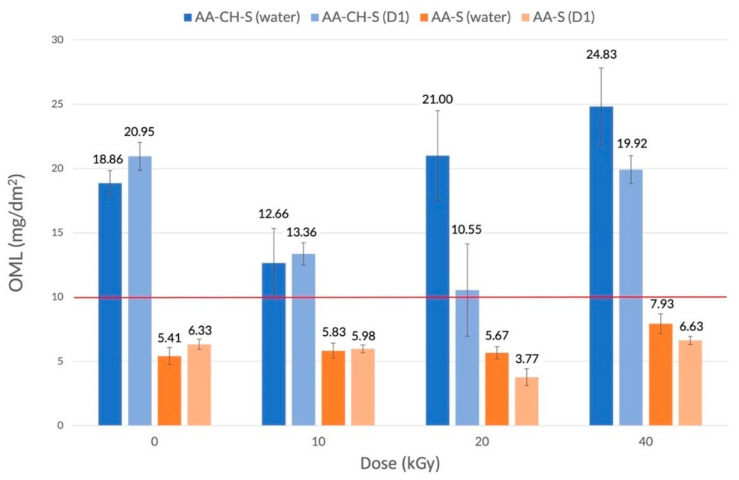
Global migration of AA-CH-S (blue) and AA-S (orange) films in two model solutions (water; D1).

The overall migration analysis revealed that in the case of the AA-CH-S sample, the migration size exceeds the permissible limit of 10 mg/dm^2^, according to the Commission Regulation (EU) No. 10/2011 [48], regardless of radiation dose; therefore, this material cannot be allowed to come into contact with hydrated food and oil in water emulsions (Figure 3). Interestingly, the allowed limit (Figure 3, red line) was also exceeded in the case of a reference sample. In the case of AA-S, the values did not exceed the acceptable level of overall migration, regardless of the radiation dose.

### 3.4. Sensory Analysis of Packaging Materials

The study allows for the assessment of the intensity of free volatile compounds, the presence of which may affect the sensory neutrality of the packaging. Packaging materials comply with FCM requirements only if they meet the limits for global, specific migration and sensory inertness. Failure to meet the requirements of even one parameter disqualifies the material as safe for FCMs. In some cases, the human senses, although burdened with many restrictions regarding the reception of stimuli, can detect a foreign flavor note, which remains on the verge of detectability of the used instrumental methods, which is why this research has become popular in the packaging industry in recent years. The results of the sensory analysis are complemented by an analysis of volatile organic compounds (VOCs), which can be derived from packaging components, printing inks, and other contaminants. Examples of such VOCs are acetone, cyclohexanone, ethanol, methyl ethyl ketone, ethyl acetate, toluene, and others.

Some food products, especially those containing significant amounts of fat, are particularly sensitive to foreign smells, which can be detected by consumers. These products, e.g., butter cookies, chocolate, almonds, and mineral water, are used as indicator products in the sensory testing of packaging materials. Sensory evaluation can be carried out according to the standardized methodologies described in the standards. The test is performed by a team trained in sensory evaluation. In the case of an analogous food evaluation team, the methods and reference substances are well-described and known. In the case of sensory analysis of packaging materials, there are no flavor patterns to train and check the sensitivity of the team. On the one hand, basic knowledge in the field of the sensory evaluation of food is used, but the key is the sensitivity to flavors and aromas associated with the production process of a given packaging material.

The tested samples did not have any foreign taste or smell (Table 2). The taste was defined as neutral or close to the neutral taste of water. The smell was imperceptible or slightly palpable. It was observed that with the increase in radiation dose, a slightly higher level of foreign taste and smell was noticeable; however, the results were at an acceptable, low level. On this basis, it can be concluded that radiation does not cause unacceptable changes in the taste or smell of the packaging materials tested.

### 3.5. Oxygen Transmission Rate

Oxygen permeability is one of the most commonly studied properties of packaging films. The tested biodegradable plastics were characterized by considerable gas permeability, i.e., low barrier properties. The OTR results for AA-S and AA-CH-S films presented in Table 3 revealed that radiation does not alter oxygen permeability. No changes in the barrier properties of the tested samples were observed, regardless of the radiation dose.

### 3.6. Water Vapor Transmission Rate

The water vapor transmission rate (WVTR) data for the AA-S and AA-CH-S films are presented in Table 4. The results showed that in the case of the AA-S sample, the radiation caused a two-fold increase in the permeability of water vapor as a result of the radiation for all the analyzed doses. Interestingly, no significant changes in water vapor permeability were observed in the case of the AA-CH-S films.

### 3.7. Color Measurements

The total color difference (Δ*E*) parameter determined for the analyzed films is presented in Table 5. The results showed that in the case of both tested materials, the magnitude of the changes correlated with the radiation dose used. However, in the case of the AA-S sample, the color changes remained at an acceptable level of Δ*E* < 1, since the observer did not notice the color difference. In the case of the AA-CH-S sample, the changes in color were more pronounced (Δ*E* < 1) only for the radiation dose of 10 kGy. For the radiation dose of 20 kGy, the changes in color can be noticed by an experienced observer, and for the radiation dose of 40 kGy, the color difference is noticeable. 

### 3.8. Structure Analysis by Fourier Transform Infrared (FTIR) Spectroscopy

**Figure 4:**
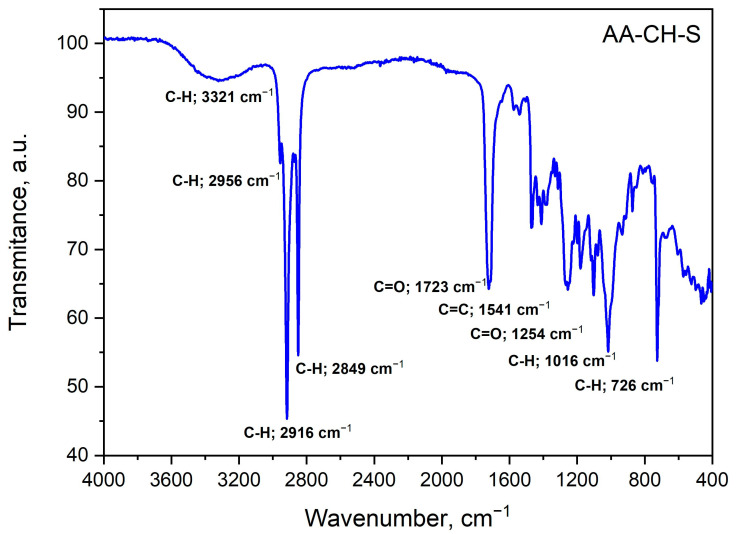
ATR FT-IR spectra of the AA-CH-S film.

The aim of the study was to characterize the changes in the structural properties of the biofilms (AA-S and AA-CH-S) during ionization irradiation with different doses. For spectral characteristics, infrared spectroscopy was used. The FT-IR spectra of the non-irradiated AA-CH-S sample are presented in Figure 4. 

The following bands were recognized in these spectra according to the literature data [62,63,64]: aromatic ring stretching vibrations of the C-H group (3321 cm^−1^); aliphatic chain stretching vibrations of the C-H groups (2956 cm^−1^); stretching vibrations from the carbonyl groups C=O (1723 cm^−1^); aromatic ring stretching vibrations of the C=C group (1541 cm^−1^). In the tested material, the presence of chitosan is most likely indicated by the band that is visible at the wavelength 2916 cm^−1^. The intensive band at 1723 cm^−1^ that is characteristic of C=O binding may be caused by the degradation of chitosan, while the medium intensity band at 1386 cm^−1^ may originate from the C-H deformation vibrations in the chitosan methylene groups. By comparing the band characteristics of polysaccharides (1200–1030 cm^−1^), pyranose rings (785–730 cm^−1^), and the spectra of a material that contained corn starch, the presence of starch in the AA-CH-S material was confirmed [62,63,64].

**Figure 5:**
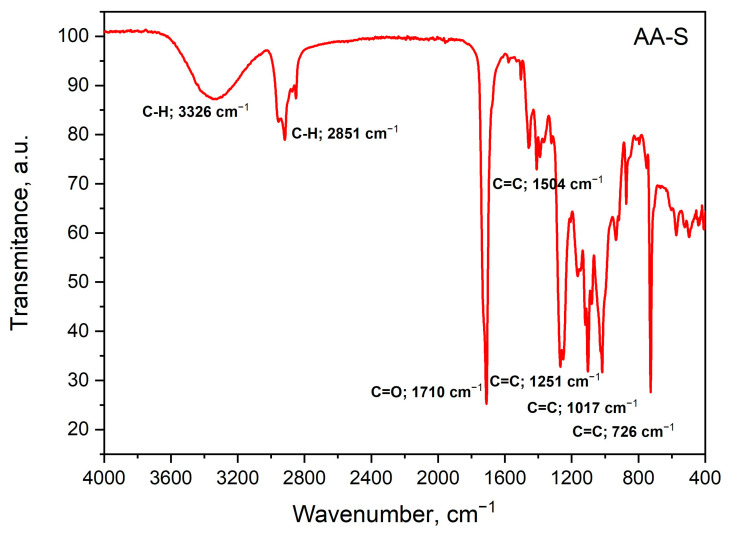
ATR FT-IR spectra of the AA-S film.

For the comparison, the mid-infrared spectra were registered and analyzed for the non-irradiated AA-S film (Figure 5). 

The following bands were recognized based on the literature data (Figure 5) [62]: aromatic ring stretching vibrations of the C-H group (3326 cm^−1^); aliphatic chain stretching vibrations of the C-H groups (2851 cm^−1^) confirmed by the presence of strong bands in the region of ester stretching vibrations of C-O (1300–1100 cm^−1^); stretching vibrations from the C=O carbonyl groups (1710 cm^−1^); aromatic ring stretching vibrations of the C=C group (1504 cm^−1^); a group of bands in the range of 785–730 cm^−1^ also found in materials that contained starch, derived from the pyranose ring of polysaccharides.

**Figure 6:**
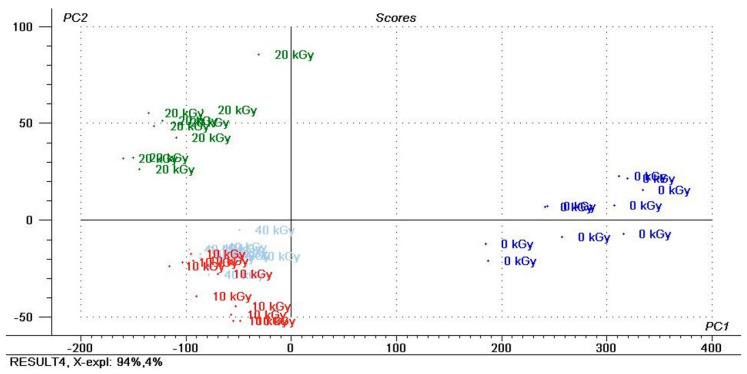
PCA results of IR spectra of the AA-CH-S film and the score plot for two significant principal components, PC1 vs. PC2 (non-irradiated (0 kGy) and irradiated with different doses (10, 20 and 40 kGy)).

**Figure 7:**
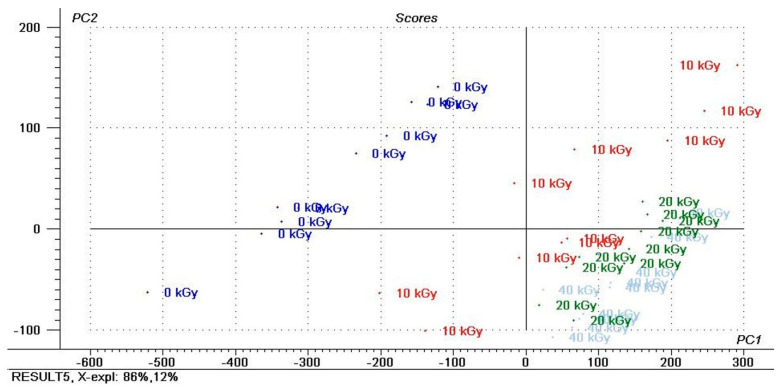
PCA results of IR spectra of the AA-S film and score plot for two significant principal components, PC1 vs. PC2 (non-irradiated (0 kGy) and irradiated with different doses (10, 20 and 40 kGy)).

Principal component analysis (PCA) was performed on the recorded infrared spectra to examine the main differences in the spectra of the examined materials irradiated by different doses of ionization radiation. Only analyses of pure spectra, without chemometrics, show no significant differences between the samples exposed to ionizing radiation. Infrared spectra can be used to distinguish reference material from radiation-treated samples, but further distinction due to the dose used is no longer possible. As a result of the PCA analysis, we could discriminate between the samples with different doses of ionization irradiation for the AA-CH-S and AA-S materials, respectively (Figure 6 and Figure 7).

The IR spectra of the AA-CH-S film irradiated by different ionization radiation doses analyzed using a PCA score plot revealed the clear separation between the samples (Figure 6). The first (PC1) and second (PC2) principal components accounted for 94.66% and 3.48% of the variance in the experimental data, respectively. The first three PCs described over 99% of the data. PC1 was strongly positively correlated with general irradiation. PC2 is dependent on the quantitative dose. Different levels of separation of the samples irradiated by 20 kGy doses in comparison to the samples irradiated by 40 kGy doses may mean irreversible changes in the structure of the material detected by IR spectroscopy.

Figure 6 presents the PCA results of the IR spectra of the AA-S material and the score plot for two significant principal components, PC1 vs. PC2. The material was non-irradiated (0 kGy) and irradiated with different doses. The IR spectra of the AA-S material irradiated by different ionization radiation doses analyzed using the PCA score plot revealed the blurred separation between the samples (Figure 7). The first (PC1) and second (PC2) principal components accounted for 85.56% and 11.58% of the variance in the experimental data, respectively. The first six principal components (PCs) described more than 99% of the data for this sample (data series). PC1 was strongly negatively correlated with the quantitative irradiation dose. 

### 3.9. Surface Morphology Using SEM Microscopy

SEM microscopic images of biobased composites after exposure to radiation was made for the surface change evaluation. The morphology of the samples’ surface is presented in Figure 8 and Figure 9. SEM images of the AA-CH-S film (Figure 8) revealed visible long structures with sharp edges, which originate from the filler chitosan. More SEM microphotographs of the AA-CH-S samples are presented in Supplementary Data in Figures S1–S18. There are no changes in the surface morphology between the reference sample (AA-CH-S 0 kGy) and the irradiated films.

The AA-S film contains starch, but its grains are not recognizable in the microscopic image and a homogenous structure can be observed. There are no visible changes or destruction in the morphology of the reference sample (AA-S 0 kGy) or the irradiated sample (Figure 9). More SEM microphotographs of the AA-S samples are presented in Supplementary Data in Figures S19–S30.

Regardless of the dose used, neither structural changes nor physical damage during the irradiation process were observed. 

### 3.10. Biodegradation in Activated Sludge Environment

**Figure 10:**
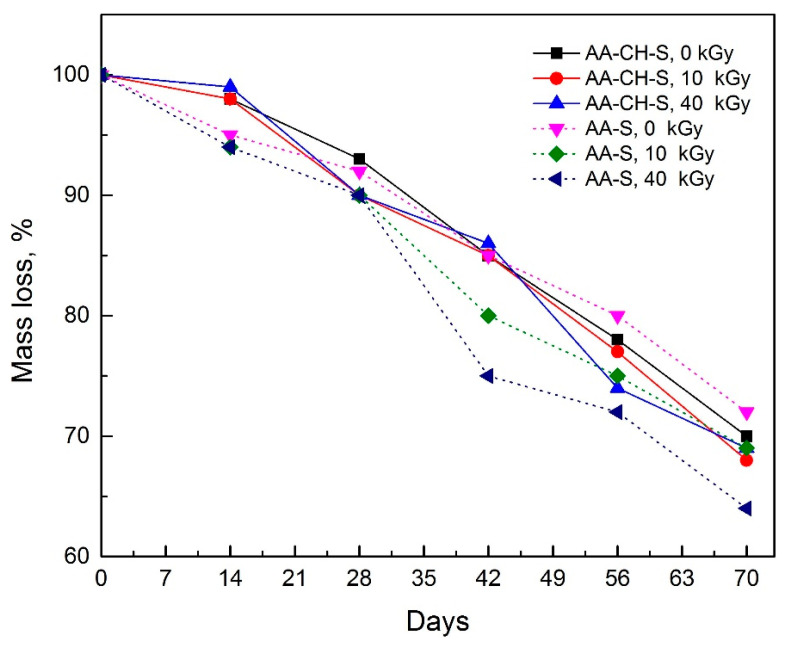
The degree of biodegradability in activated sludge was measured by the mass loss of the AA-S and AA-CH-S films exposed to 10 and 40 kGy radiation doses.

Biodegradation is a specific process that takes place in stages and depends on many external factors, including the characteristics of the material, pH, and humidity of the environment, temperature, and types of microorganisms. This work involved pilot studies on biodegradation in activated sludge conditions for the samples irradiated with a radiation dose of 10 and 40 kGy, and a reference material (Figure 10). The results showed that in the case of the AA-S samples, the higher the radiation dose, the faster the biodegradation rate. In the case of the AA-CH-S material, the radiation did not affect the biodegradation process.

## 4. Conclusions

This paper presents the results of the tests that were carried out to determine the changes in the biodegradable materials AA-CH-S and AA-S caused by gamma radiation (10, 20, and 40 kGy exposure doses). The results showed that the mechanical properties of AA-S were improved due to the radiation-induced cross-linking processes, while in the case of AA-CH-S, a considerable decrease in the elongation at break was observed. The results also showed a decrease in the WVTR in the case of AA-S and no changes in barrier properties in the case of AA-CH-S. Both materials revealed no changes in the odor analyzed by the sensory analysis. 

Our tests attempt to determine the biodegradability of the tested materials in a liquid environment. It has been shown that ionizing radiation, in the range of the radiation doses applied, affected only the AA-S material. Furthermore, an important aspect of the research was also the evaluation of the packaging–product interaction through the study of overall migration, which determines the suitability of the packaging material for contact with the packaged products, particularly food. The results revealed the unacceptable overall migration of AA-CH-S. Our study showed that for new packing system development based on natural material composites, evaluation of the radiation impact should be intensively studied before final product implementation. 

## Figures and Tables

**Figure 8:**
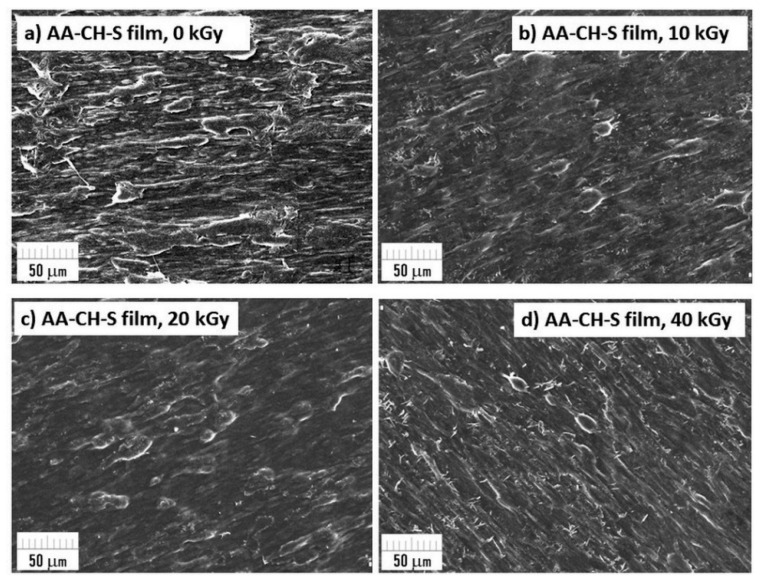
SEM micrographs of AA-CH-S films (0, 10, 20; 40 kGy) taken at 500× magnification.

**Figure 9:**
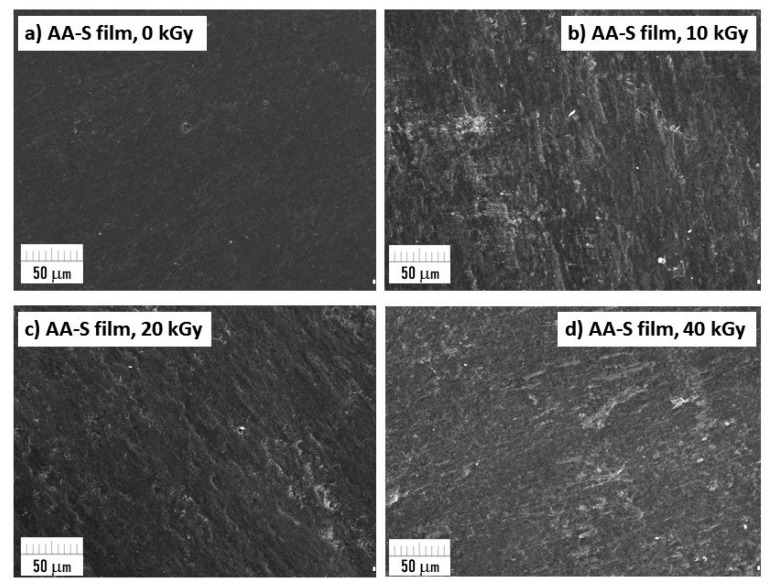
SEM micrographs of AA-S films (0, 10, 20; 40 kGy) taken at 500× magnification.

**Table 1:** Samples’ description.

Sample	Characteristics	Symbol
Aliphatic–aromatic copolyester with chitosan and thermoplastic starch film	Flexible film, milky, translucent, food contact material; industrially compostable (about 14 days)	AA-CH-S
Aliphatic–aromatic copolyester with thermoplastic starch film	Flexible film, creamy yellow, food contact material; industrially compostable	AA-S

**Table 2:** Sensory analysis of AA-CH-S and AA-S films.

Dose (kGy)	AA-CH-S Flavor/Odor Median	AA-S Flavor/Odor Median
0	0.0/0.0	0.0/0.0
10	0.0/0.0	0.0/0.0
20	0.5/1.0	0.0/0.5
40	0.5/1.0	0.0/1.0

**Table 3:** OTR (cc/m^2^/24 h) results of AA-S and AA-CH-S films.

Dose (kGy)	AA-CH-S	AA-S
0	85 ± 10	120 ± 15
10	80 ± 5	115 ± 5
20	85 ± 8	120 ± 10
40	90 ± 10	115 ± 10

**Table 4:** WVTR (g·m^−2^·d^−1^) results of AA-S and AA-CH-S films.

Dose (kGy)	AA-CH-S	AA-S
0	300 ± 30	110 ± 15
10	340 ± 30	210 ± 10
20	300 ± 40	220 ± 5
40	280 ± 10	210 ± 10

**Table 5:** The average total color difference (Δ*E*) results for the analyzed films.

Dose (kGy)	AA-CH-S	AA-S
10	0.77	0.21
20	1.62	0.28
40	3.79	0.36

## Data Availability

Not applicable.

## Supplementary Materials

The following are available online at https://www.mdpi.com/article/10.3390/ma16020859/s1, Figures S1–S18: selected SEM micrographs of AA-CH-S films taken at different magnification; Figures S19–S30 selected SEM micrographs of AA-S films taken at different magnification.

## References

[1] Tsang Y.F., Kumar V., Samadar P., Yang Y., Lee J., Ok Y.S., Song H., Kim K.H., Kwon E.E., Jeon Y.J. (2019). Production of Bioplastic through Food Waste Valorization. Environ. Int..

[2] Delgado M., Felix M., Bengoechea C. (2018). Development of Bioplastic Materials: From Rapeseed Oil Industry by Products to Added-Value Biodegradable Biocomposite Materials. Ind. Crops Prod..

[3] Bilo F., Pandini S., Sartore L., Depero L.E., Gargiulo G., Bonassi A., Federici S., Bontempi E. (2018). A Sustainable Bioplastic Obtained from Rice Straw. J. Clean. Prod..

[4] Rajesh Banu J., Kavitha S., Yukesh Kannah R., Poornima Devi T., Gunasekaran M., Kim S.H., Kumar G. (2019). A Review on Biopolymer Production via Lignin Valorization. Bioresour. Technol..

[5] European Bioplastics Materials—European Bioplastics e.V. https://www.european-bioplastics.org/bioplastics/materials/.

[6] European Bioplstics Bioplastic Facts and Figures. https://docs.european-bioplastics.org/publications/EUBP_Facts_and_figures.pdf.

[7] Yu L., Dean K., Li L. (2006). Polymer Blends and Composites from Renewable Resources. Prog. Polym. Sci..

[8] Assman K., Tomasz K. (2017). Polimery Biodegradowalne - Przykłady Zastosowań. Nowoczesne Materiały Polimerowe I Ich Przetwórstwo.

[9] Czarnecka-Komorowska D., Tomasik M., Thakur V.K., Kostecka E., Rydzkowski T., Jursa-Kulesza J., Bryll K., Mysłowski J., Gawdzińska K. (2022). Biocomposite Composting Based on the Sugar-Protein Condensation Theory. Ind. Crops Prod..

[10] Kraśniewska K., Pobiega K., Gniewosz M. (2019). Pullulan-Biopolymer with Potential for Use as Food Packaging. Int. J. Food Eng..

[11] Acha C., Blanchard R., Brodsky J., Ding L., Fox A., Grosvenor E., Gibson K., Hoy A., Hughes J., Lee K. (2022). On the Mechanism of Electron Beam Radiation-Induced Modification of Poly(Lactic Acid) for Applications in Biodegradable Food Packaging. Appl. Sci..

[12] Abramowska A., Cieśla K.A. (2021). The Influence of Electron and Gamma Irradiation on the Properties of Starch:PVA Films—The Effect of Irradiation Dose. Nukleonika.

[13] Madera-Santana T.J., Meléndrez R., González-García G., Quintana-Owen P., Pillai S.D. (2016). Effect of Gamma Irradiation on Physicochemical Properties of Commercial Poly(Lactic Acid) Clamshell for Food Packaging. Radiat. Phys. Chem..

[14] Li L., Chen H., Wang M., Lv X., Zhao Y., Xia L. (2018). Development and Characterization of Irradiated-Corn-Starch Films. Carbohydr. Polym..

[15] Díez-Pascual A.M. (2019). Synthesis and Applications of Biopolymer Composites. Int. J. Mol. Sci..

[16] Salapare H.S., Amigoni S., Guittard F. (2019). Bioinspired and Biobased Materials. Macromol. Chem. Phys..

[17] van den Oever M., Molenveld K. (2017). Replacing Fossil Based Plastic Performance Products by Bio-Based Plastic Products-Technical Feasibility. New Biotechnol..

[18] Habel C., Schöttle M., Daab M., Eichstaedt N.J., Wagner D., Bakhshi H., Agarwal S., Horn M.A., Breu J., Habel C. (2018). High-Barrier, Biodegradable Food Packaging. Macromol. Mater. Eng..

[19] Sangroniz A., Sangroniz L., Gonzalez A., Santamaria A., del Rio J., Iriarte M., Etxeberria A. (2019). Improving the Barrier Properties of a Biodegradable Polyester for Packaging Applications. Eur. Polym. J..

[20] Aniśko J., Barczewski M. (2021). Polylactide: From Synthesis and Modification to Final Properties. Adv. Sci. Technol. Res. J..

[21] Peelman N., Ragaert P., de Meulenaer B., Adons D., Peeters R., Cardon L., van Impe F., Devlieghere F. (2013). Application of Bioplastics for Food Packaging. Trends Food Sci. Technol..

[22] Ruggero F., Gori R., Lubello C. (2019). Methodologies to Assess Biodegradation of Bioplastics during Aerobic Composting and Anaerobic Digestion: A Review. Waste Manag. Res..

[23] Abramowska A., Cieśla K.A., Buczkowski M.J., Nowicki A., Głuszewski W. (2015). The Influence of Ionizing Radiation on the Properties of Starch-PVA Films. Nukleonika.

[24] Flores I., Martínez De Ilarduya A., Sardon H., Müller A.J., Muñoz-Guerra S. (2019). Synthesis of Aromatic-Aliphatic Polyesters by Enzymatic Ring Opening Polymerization of Cyclic Oligoesters and Their Cyclodepolymerization for a Circular Economy. ACS Appl. Polym. Mater..

[25] Ukielski R., Kondratowicz F., Kotowski D. (2013). Production, Properties and Trends in Development of Biodegradable Polyesters with Particular Respect to Aliphatic-Aromatic Copolymers/Produkcja, Wlasciwosci i Kierunki Rozwoju Biodegradowalnych Poliestrow Ze Szczegolnym Uwzglednieniem Kopolimerow Alifatyczno-Aromatycznych. Polimery.

[26] Chandra R., Rustgi R. (1998). Biodegradable Polymers. Prog. Polym. Sci..

[27] Witt U., Müller R.J., Deckwer W.D. (1997). Biodegradation Behavior and Material Properties of Aliphatic/Aromatic Polyesters of Commercial Importance. J. Environ. Polym. Degrad..

[28] Kubera H., Assman K., Czaja-Jagielska N., Melski K., Głuszewski W., Migdał W., Zimek Z. (2012). Impact of Ionizing Radiation on the Properties of a Hydrobiodegradable Aliphatic-Aromatic Copolyester. Nukleonika.

[29] Khan B., Bilal Khan Niazi M., Samin G., Jahan Z. (2017). Thermoplastic Starch: A Possible Biodegradable Food Packaging Material—A Review. J. Food Process. Eng..

[30] Knitter M., Czarnecka-Komorowska D., Czaja-Jagielska N., Szymanowska-Powałowska D. (2019). Manufacturing and Properties of Biodegradable Composites Based on Thermoplastic Starch/Polyethylene-Vinyl Alcohol and Silver Particles. International Scientific-Technical Conference MANUFACTURING.

[31] Nadia N., Othman S.A. (2019). Gamma Radiation Effects on Biodegradable Starch Based Blend With Different Polyester: A Review. J. Adv. Res. Dyn. Control Syst..

[32] Guo Y., Wang H. (2019). Preparation and Properties of Edible Packaging Films Based on Chitosan with Microcrystalline Cellulose from Tomato Peel Pomace. J. Biobased Mater. Bioenergy.

[33] Ahmed K.B.M., Khan M.M.A., Siddiqui H., Jahan A. (2020). Chitosan and Its Oligosaccharides, a Promising Option for Sustainable Crop Production—A Review. Carbohydr. Polym..

[34] Dong Z., Cui H., Wang Y., Wang C., Li Y., Wang C. (2020). Biocompatible AIE Material from Natural Resources: Chitosan and Its Multifunctional Applications. Carbohydr. Polym..

[35] Malinowska-Pańczyk E., Sztuka K., Kołodziejska I. (2010). Substancje o Działaniu Przeciwdrobnoustrojowym Jako Składniki Biodegradowalnych Folii z Polimerów Naturalnych. Polimery.

[36] Rinaudo M. (2006). Chitin and Chitosan: Properties and Applications. Prog. Polym. Sci..

[37] Michieletto A., Lorandi F., de Bon F., Isse A.A., Gennaro A. (2020). Biocompatible Polymers via Aqueous Electrochemically Mediated Atom Transfer Radical Polymerization. J. Polym. Sci..

[38] Madni A., Kousar R., Naeem N., Wahid F. (2021). Recent Advancements in Applications of Chitosan-Based Biomaterials for Skin Tissue Engineering. J. Bioresour. Bioprod..

[39] Saad E.M., Elshaarawy R.F., Mahmoud S.A., El-Moselhy K.M. (2021). New Ulva Lactuca Algae Based Chitosan Bio-Composites for Bioremediation of Cd(II) Ions. J. Bioresour. Bioprod..

[40] Srinivasa P.C., Ramesh M.N., Tharanathan R.N. (2007). Effect of Plasticizers and Fatty Acids on Mechanical and Permeability Characteristics of Chitosan Films. Food Hydrocoll..

[41] Sindhu M., Abraham T. (2008). Emilia Characterisation of Ferulic Acid Incorporated Starch–Chitosan Blend Films. Food Hydrocoll..

[42] Komolprasert V. (2016). Packaging Food for Radiation Processing. Radiat. Phys. Chem..

[43] Eyssa H.M., Sawires S.G., Senna M.M. (2019). Gamma Irradiation of Polyethylene Nanocomposites for Food Packaging Applications against Stored-Product Insect Pests. J. Vinyl Addit. Technol..

[44] Silvestre C., Cimmino S., Stoleru E., Vasile C., Yongxia S., Andrzej G.C. (2017). Application of Radiation Technology to Food Packaging. Applications of Ionizing Radiation in Materials Processing.

[45] Jedson A., Brant C., Naime N., Lugão A.B., Ponce P. (2018). Influence of Ionizing Radiation on Biodegradable Foam Trays for Food Packaging Obtained from Irradiated Cassava Starch. Braz. Arch. Biol. Technol..

[46] Negrin M., Macerata E., Consolati G., Quasso F., Genovese L., Soccio M., Giola M., Lotti N., Munari A., Mariani M. (2018). Gamma Radiation Effects on Random Copolymers Based on Poly(Butylene Succinate) for Packaging Applications. Radiat. Phys. Chem..

[47] (2004). Regulation (EC) No 1935/2004 of the European Parliament and of the Council of 27 October 2004 on Materials and Articles Intended to Come into Contact with Food and Repealing Directives 80/590/EEC and 89/109/EEC.

[48] (2011). Commission Regulation (EU) No 10/2011 of 14 January 2011 on Plastic Materials and Articles Intended to Come into Contact with Food.

[49] (2006). Commission Regulation (EC) No 2023/2006 of 22 December 2006 on Good Manufacturing Practice for Materials and Articles Intended to Come into Contact with Food.

[50] Drobny J.G. (2012). Ionizing Radiation and Polymers: Principles, Technology, and Applications. Ionizing Radiation and Polymers: Principles, Technology, and Applications.

[51] Hara M. (2022). Effects of Ionizing Radiation on Biopolymers for Applications as Biomaterials. Biomed. Mater. Devices.

[52] (2019). Plastics—Determination of Tensile Properties—Part 1: General Principles.

[53] (2018). Plastics—Determination of Tensile Properties—Part 3: Test Conditions for Films and Sheets.

[54] (2002). Materials and Articles in Contact with Foodstuffs—Plastics—Part 1: Guide to The Selection of Conditions and Test Methods for Overall Migration.

[55] (2002). Materials and Articles in Contact with Foodstuffs—Plastics—Part 3: Test Methods for Overall Migration in Evaporable Simulants.

[56] (2004). Sensory Analysis—Testing of Packaging Materials and Packaging Materials and Packages for Food Products.

[57] (2022). Standard Test Method for Oxygen Gas Transmission Rate through Plastic Film and Sheeting Using a Dynamic Accumulation Method.

[58] (2021). Standard Test Method for Oxygen Headspace Analysis of Packages Using Fluorescent Decay.

[59] (2022). Standard Test Methods for Gravimetric Determination of Water Vapor Transmission Rate of Materials.

[60] Shi A.M., Wang L.J., Li D., Adhikari B. (2013). Characterization of Starch Films Containing Starch Nanoparticles: Part 1: Physical and Mechanical Properties. Carbohydr. Polym..

[61] Chorobiński M., Skowroński Ł., Bieliński M. (2019). Methodology for Determining Selected Characteristics of Polyethylene Dyeing Using CIELab System. Polimery.

[62] Silverstein R.M., Webster F.X., Kiemle D.J., Bryce D.L. (2014). Spectrometric Identification of Organic Compounds.

[63] Paluszkiewicz C., Stodolak E., Hasik M., Blazewicz M. (2011). FT-IR Study of Montmorillonite-Chitosan Nanocomposite Materials. Spectrochim. Acta Part A Mol. Biomol. Spectrosc..

[64] Silva S.M.L., Braga C.R.C., Fook M.V.L., Raposo C.M.O., Carvalho L.H., Canedo E.L., Theophile T. (2012). Application of Infrared Spectroscopy to Analysis of Chitosan/Clay Nanocomposites. Infrared Spectroscopy: Materials Science, Engineering and Technology.

