# Supplementary material for: Characterization of Biodegradable Food Contact Materials under Gamma-Radiation Treatment
Source: Materials (Basel). 2023 Jan 16;16(2):859. doi: 10.3390/ma16020859 (PMC9861635; doi:10.3390/ma16020859)

Figure S1. SEM micrographs of AA-CH-S films taken at different magnification

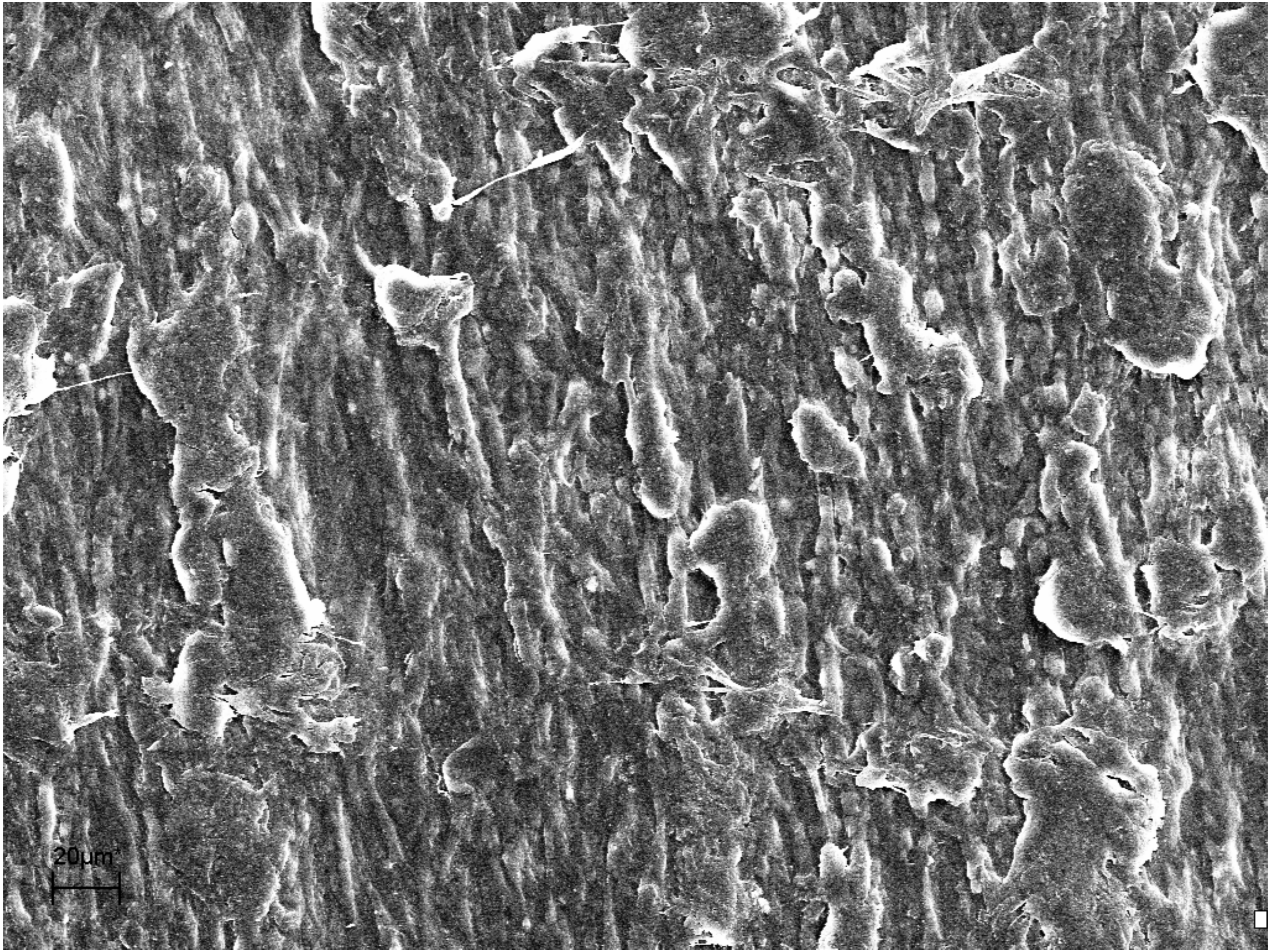

Figure S2. SEM micrographs of AA-CH-S films taken at different magnification

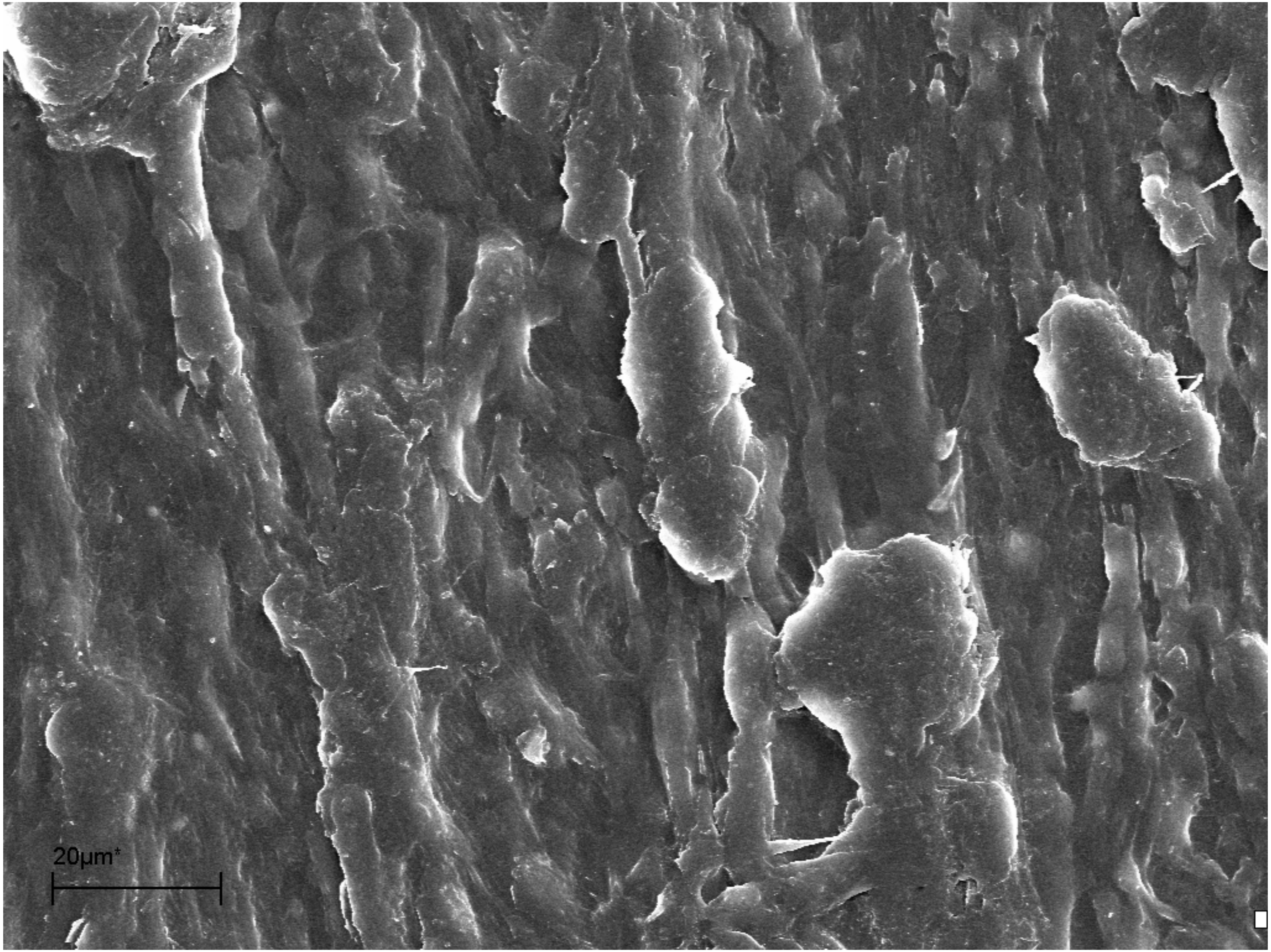

Figure S3. SEM micrographs of AA-CH-S films taken at different magnification

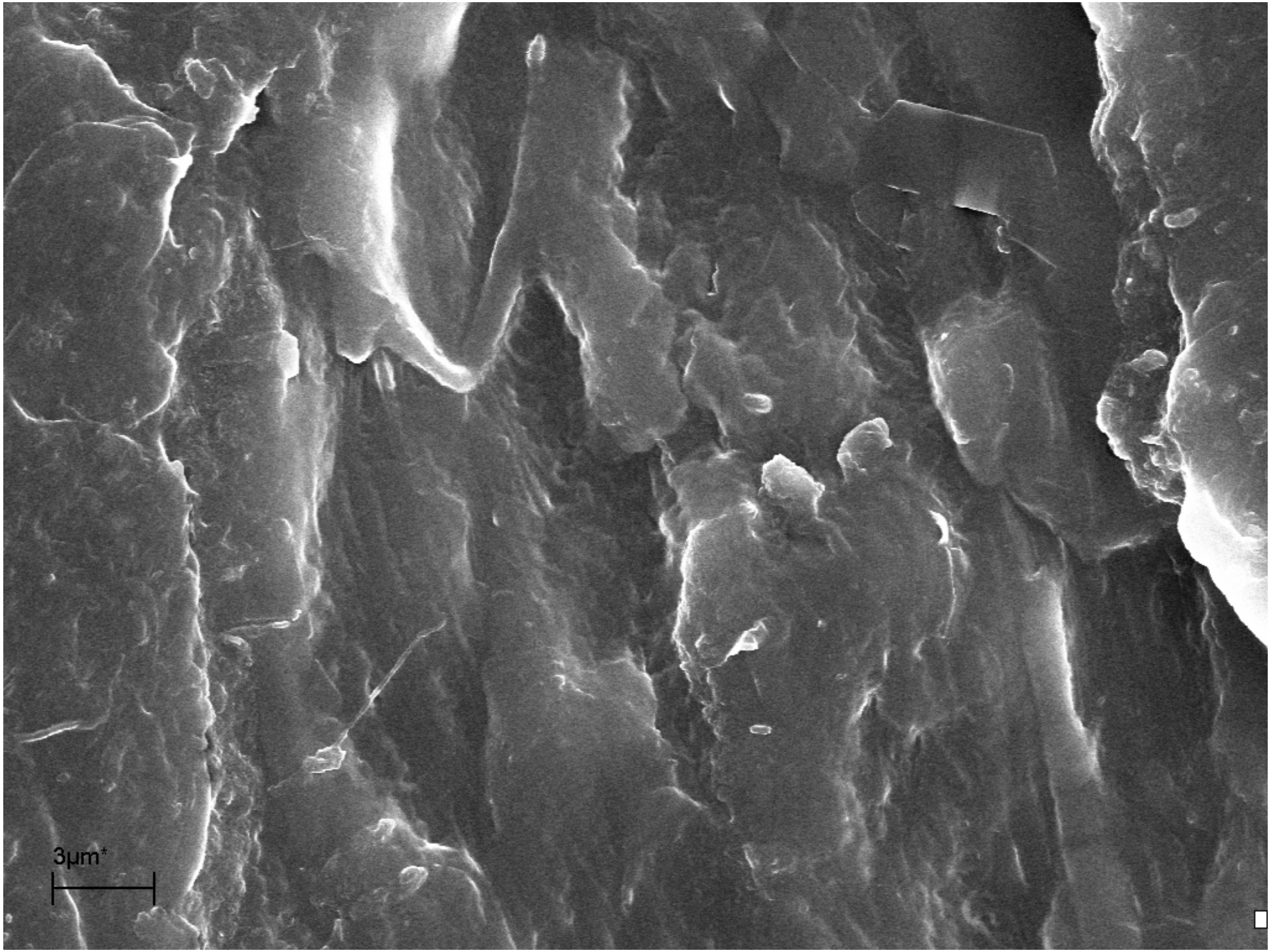

Figure S4. SEM micrographs of AA-CH-S films under 10 kGy gamma radiation dose taken at different magnification

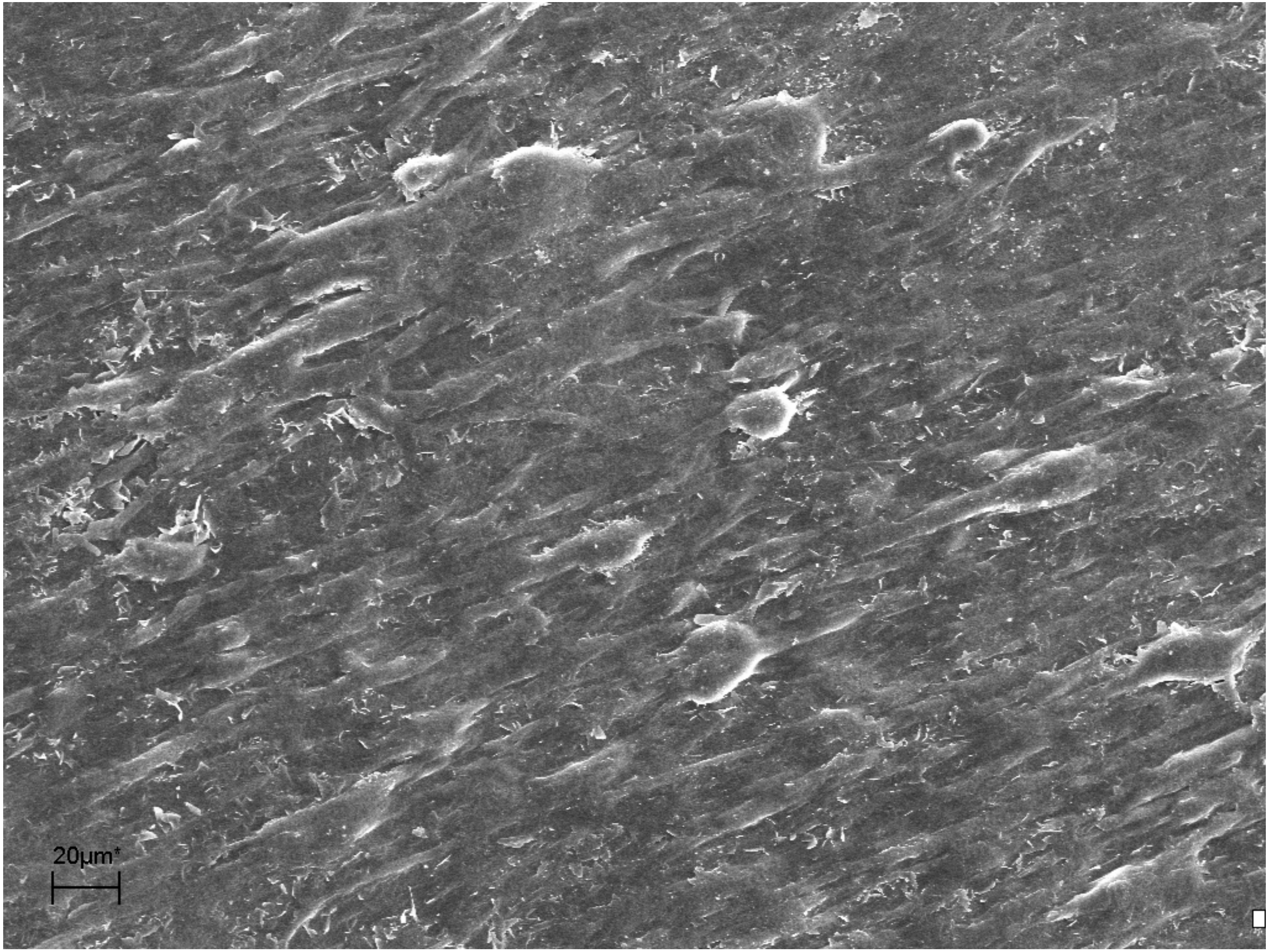

Figure S5. SEM micrographs of AA-CH-S films under 10 kGy gamma radiation dose taken at different magnification

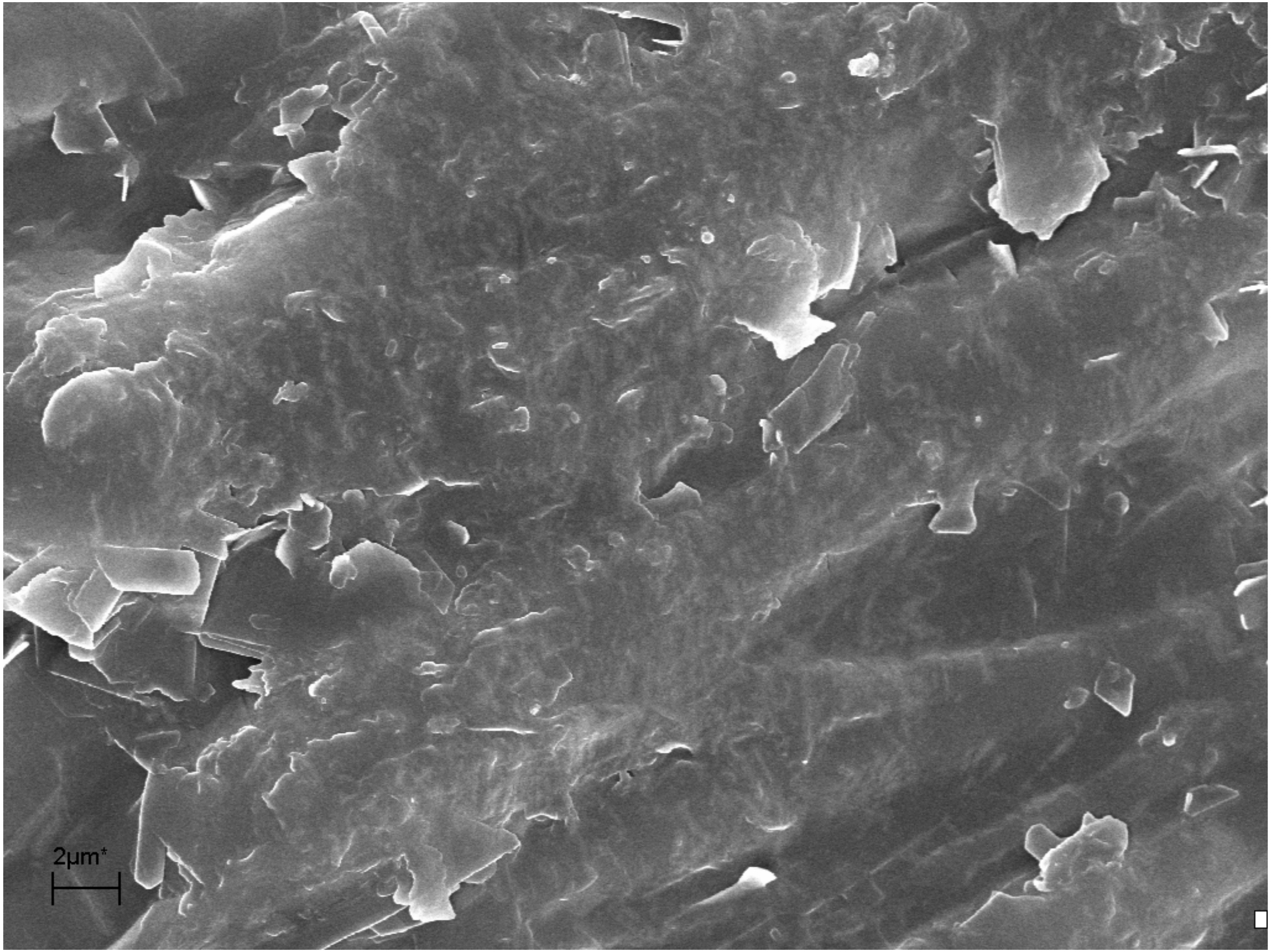

Figure S6. SEM micrographs of AA-CH-S films under 10 kGy gamma radiation dose taken at different magnification

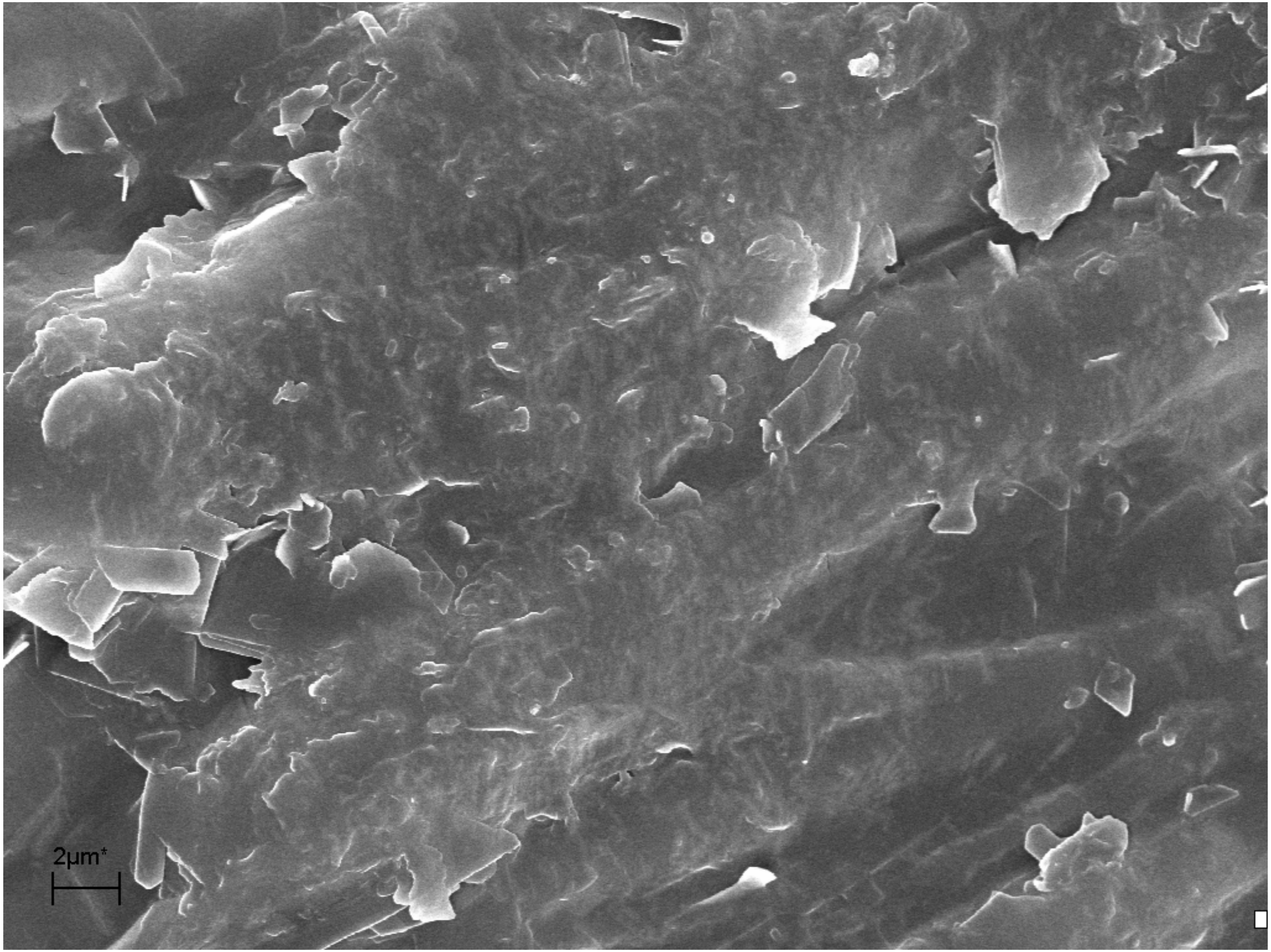

Figure S7. SEM micrographs of AA-CH-S films under 10 kGy gamma radiation dose taken at different magnification

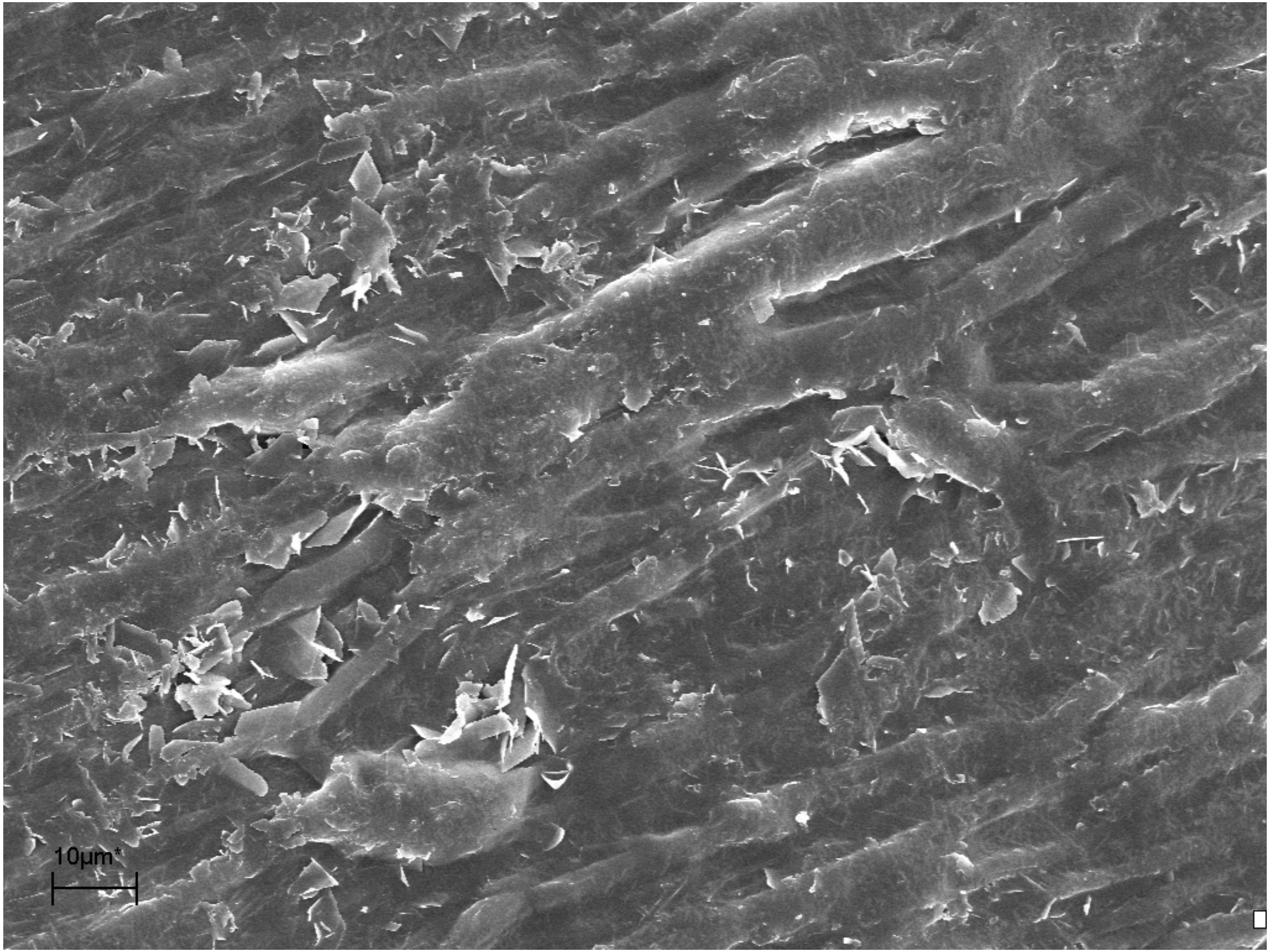

Figure S8. SEM micrographs of AA-CH-S films under 10 kGy gamma radiation dose taken at different magnification

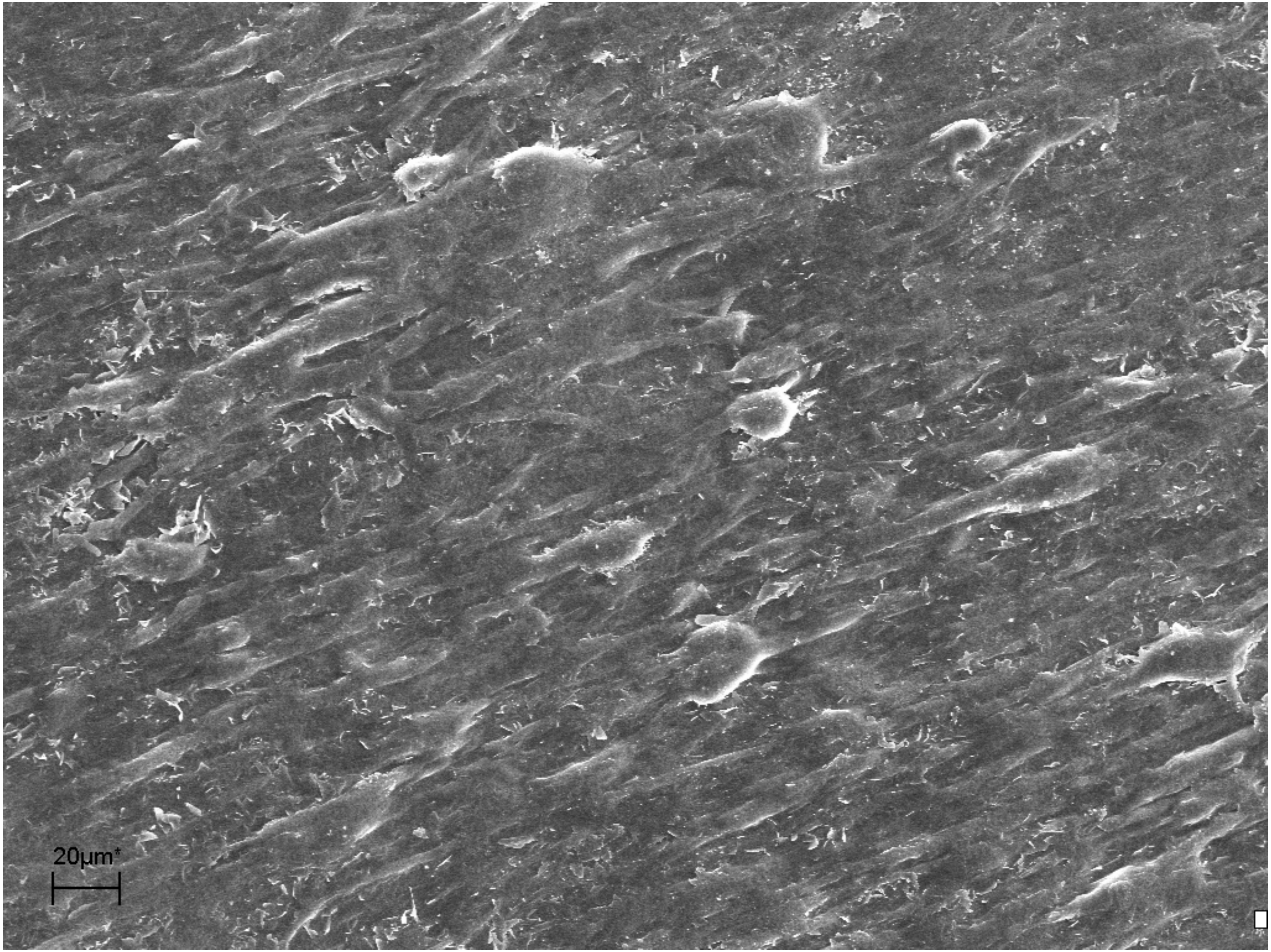

Figure S9. SEM micrographs of AA-CH-S films under 20 kGy gamma radiation dose taken at different magnification

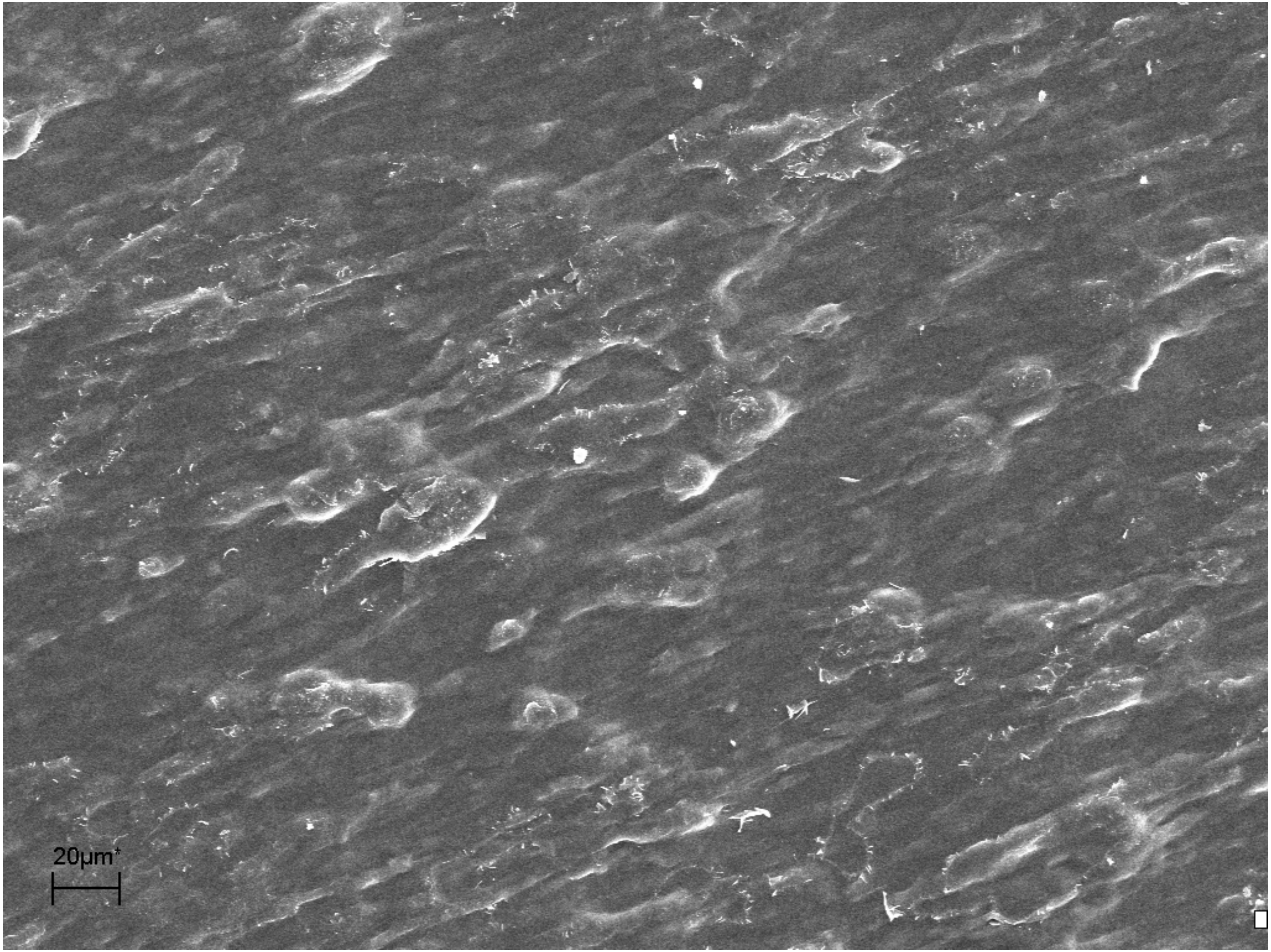

Figure S10. SEM micrographs of AA-CH-S films under 20 kGy gamma radiation dose taken at different magnification

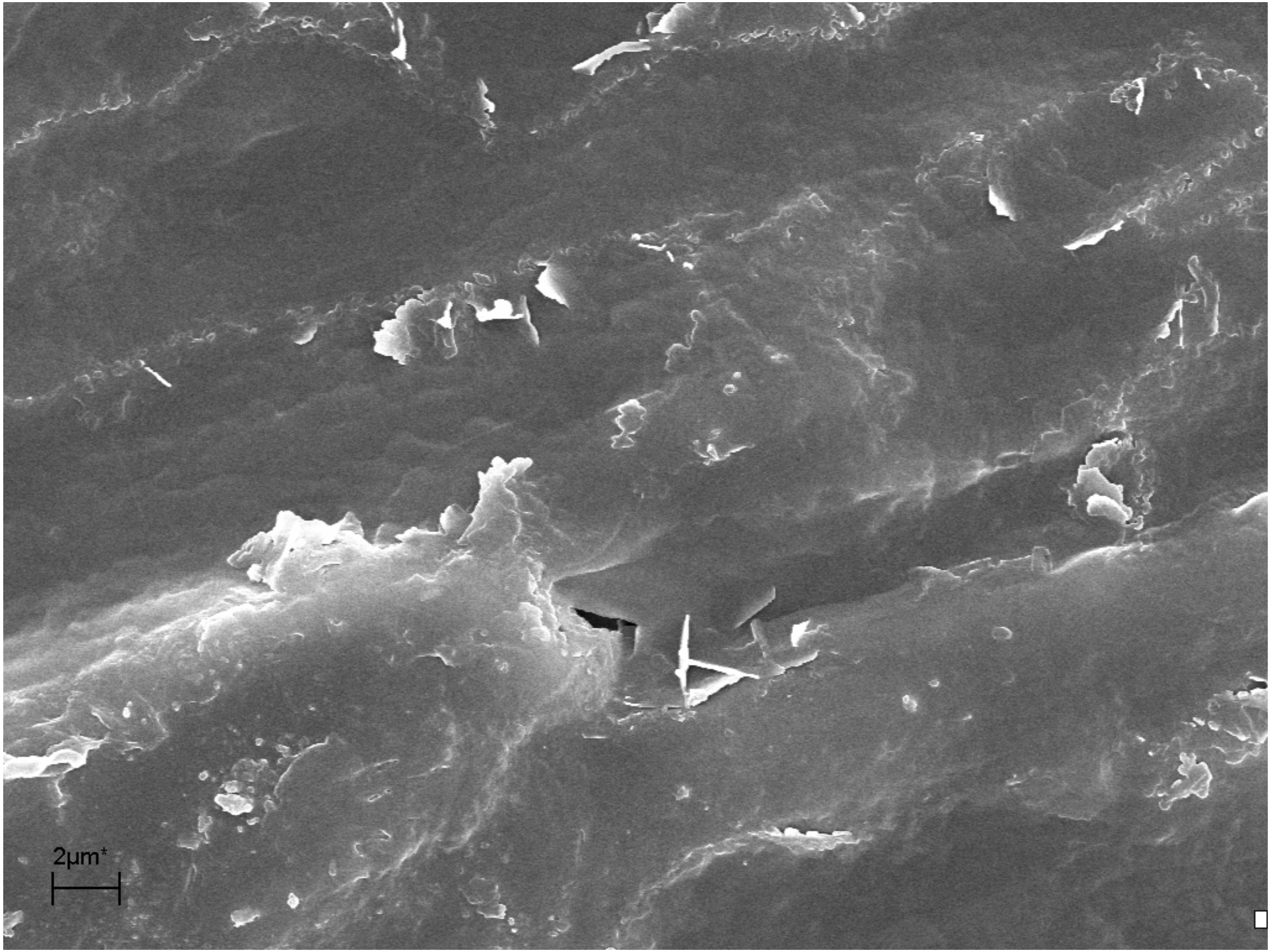

Figure S11. SEM micrographs of AA-CH-S films under 20 kGy gamma radiation dose taken at different magnification

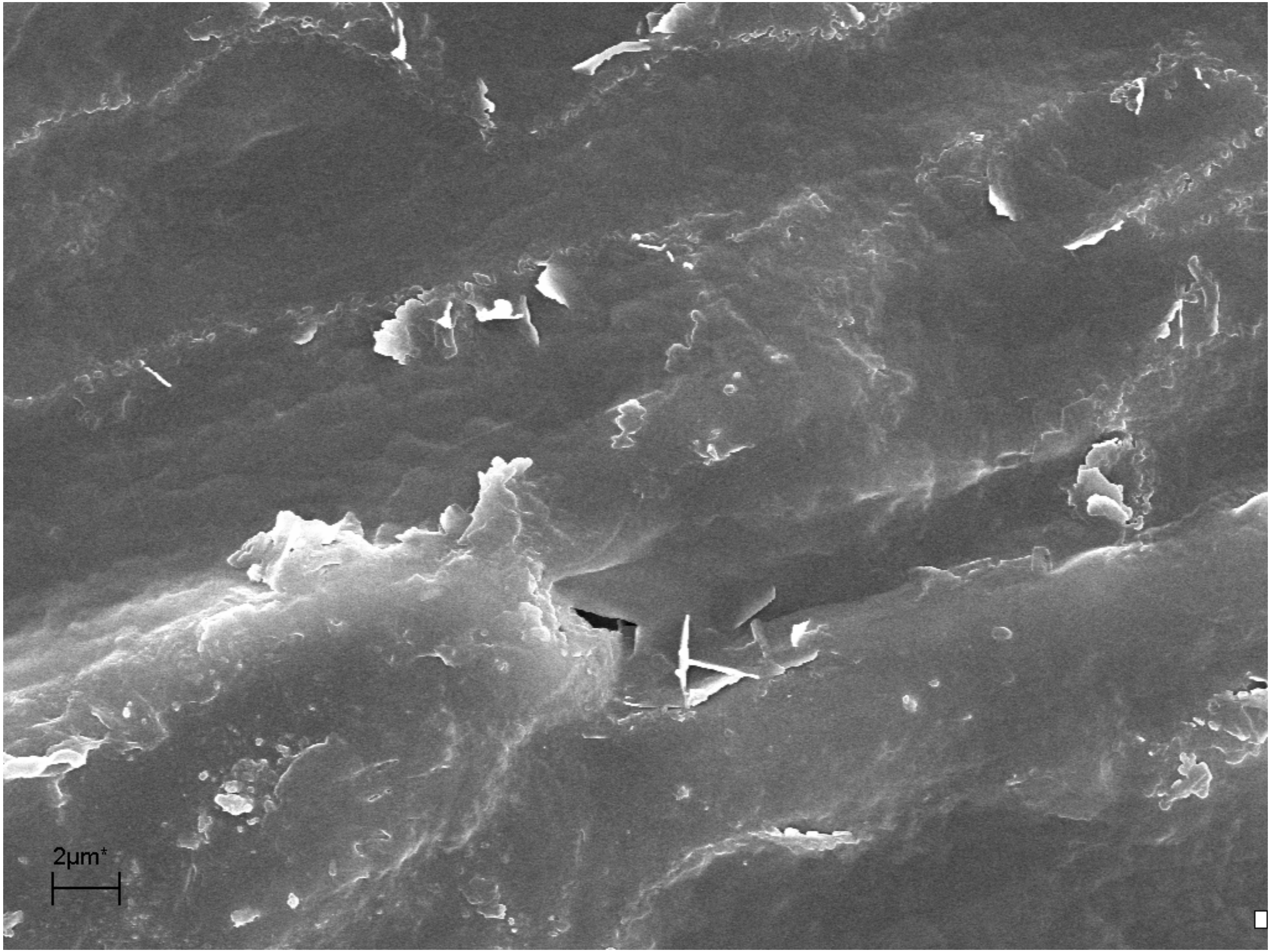

Figure S12. SEM micrographs of AA-CH-S films under 20 kGy gamma radiation dose taken at different magnification

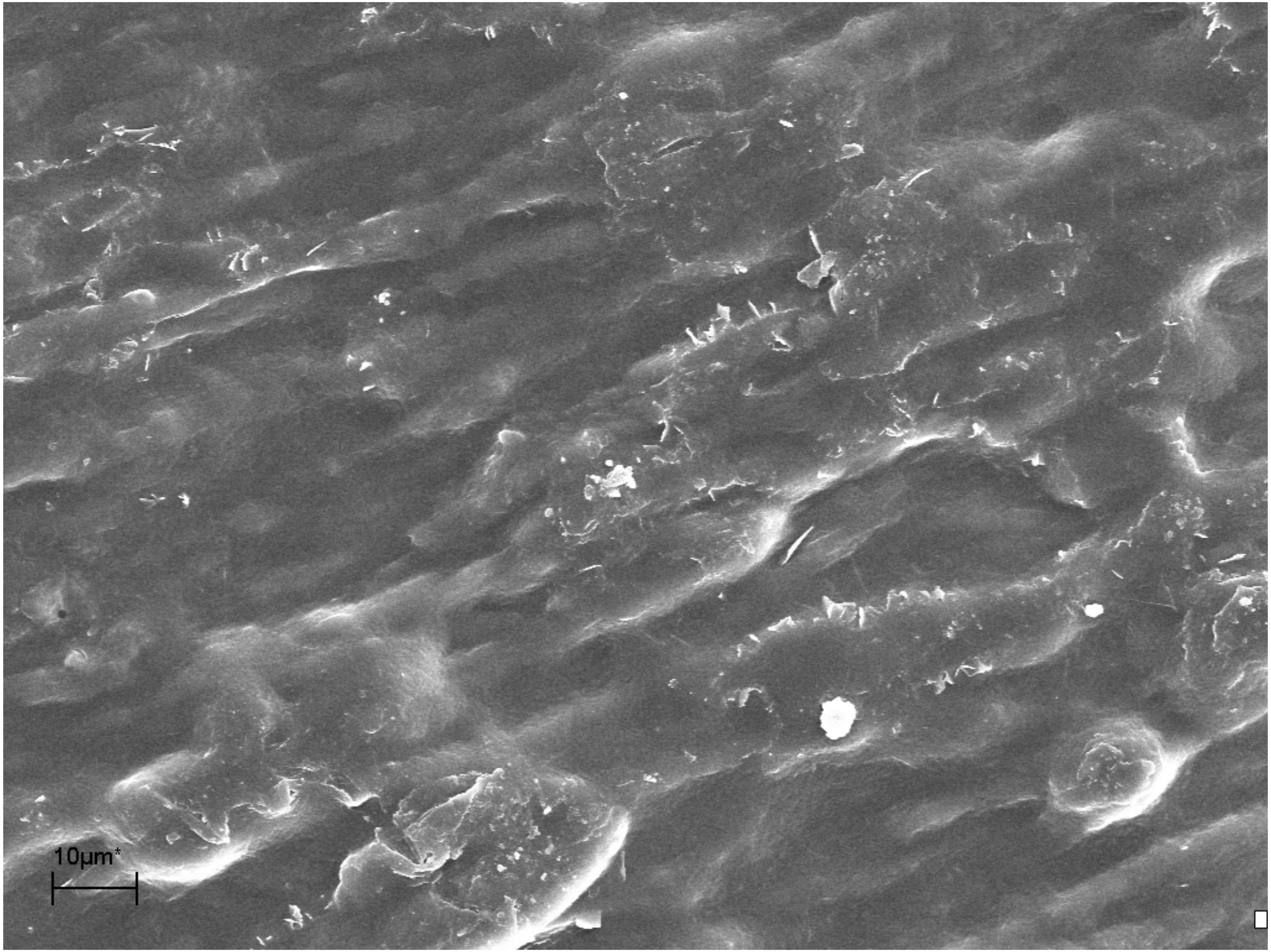

Figure S13. SEM micrographs of AA-CH-S films under 20 kGy gamma radiation dose taken at different magnification

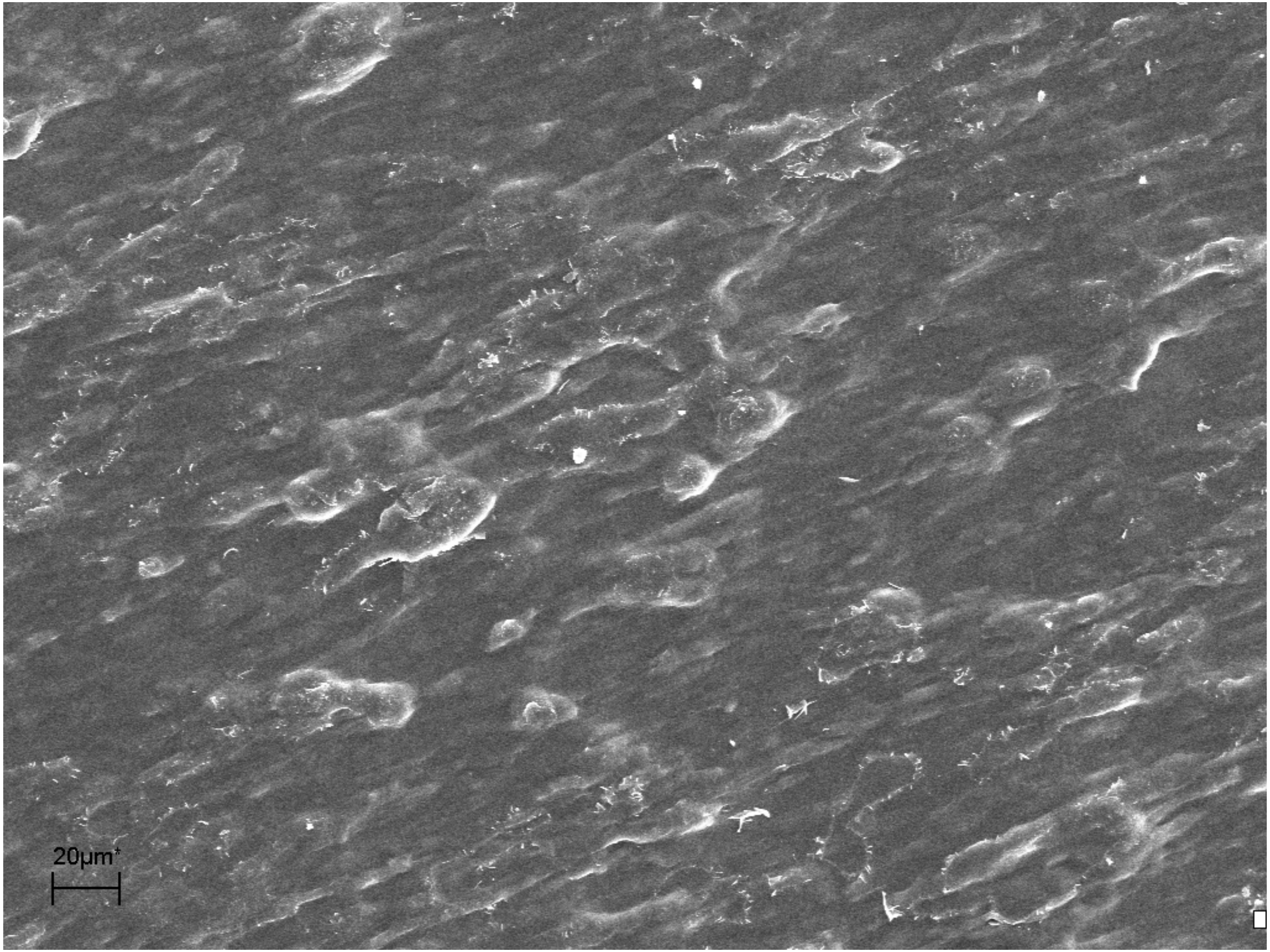

Figure S14. SEM micrographs of AA-CH-S films under 40 kGy gamma radiation dose taken at different magnification

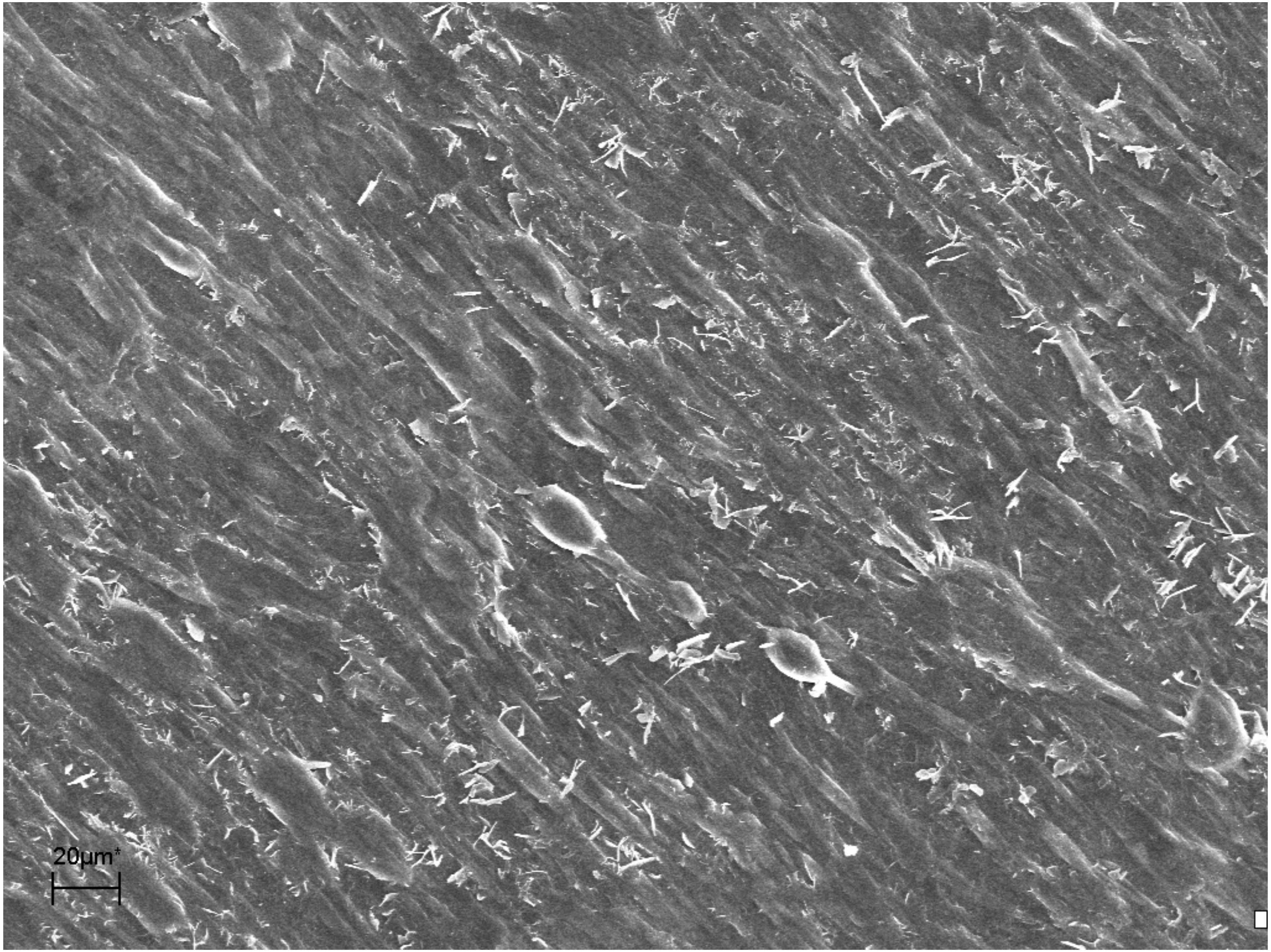

Figure S15. SEM micrographs of AA-CH-S films under 40 kGy gamma radiation dose taken at different magnification

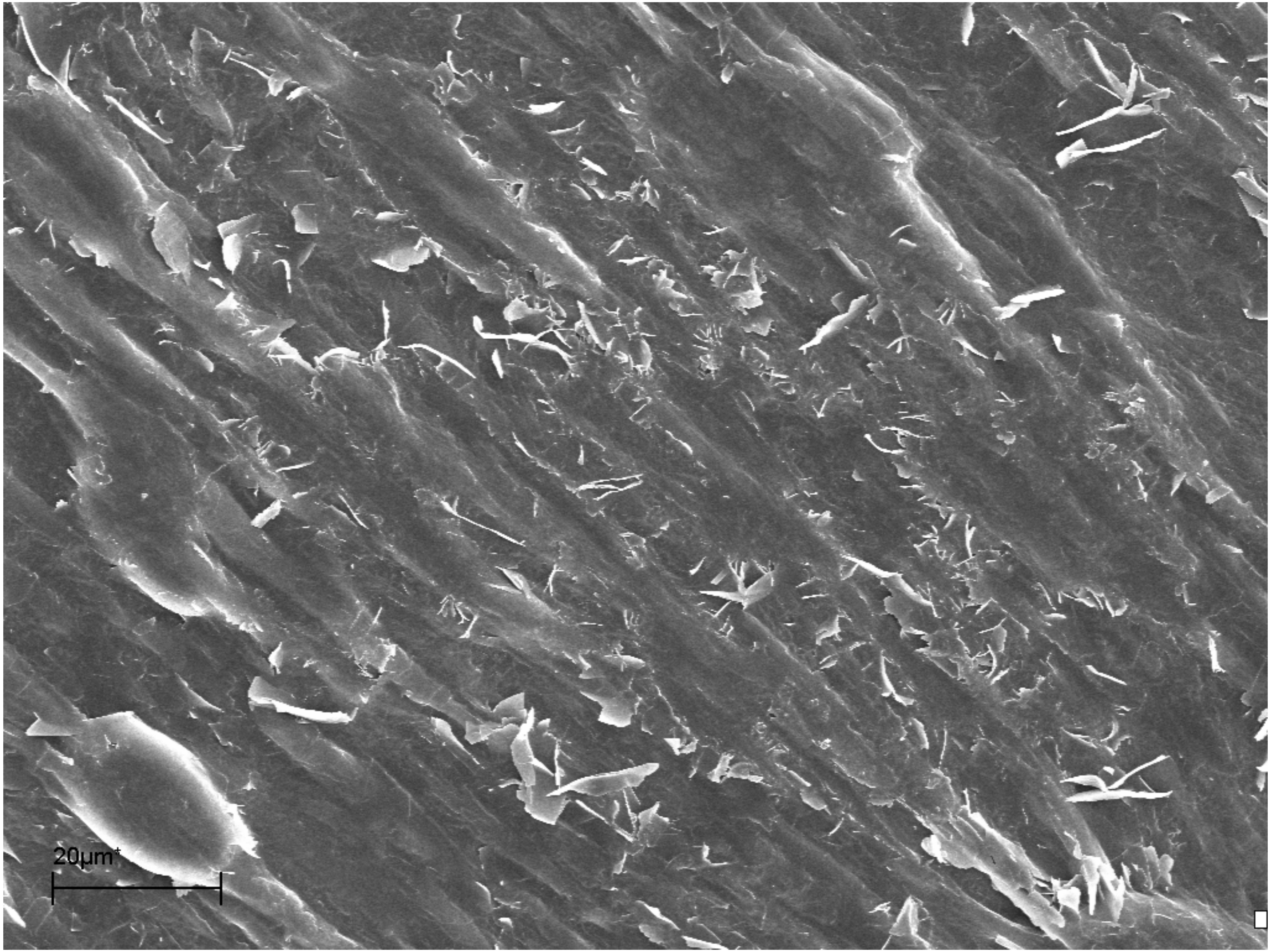

Figure S16. SEM micrographs of AA-CH-S films under 40 kGy gamma radiation dose taken at different magnification

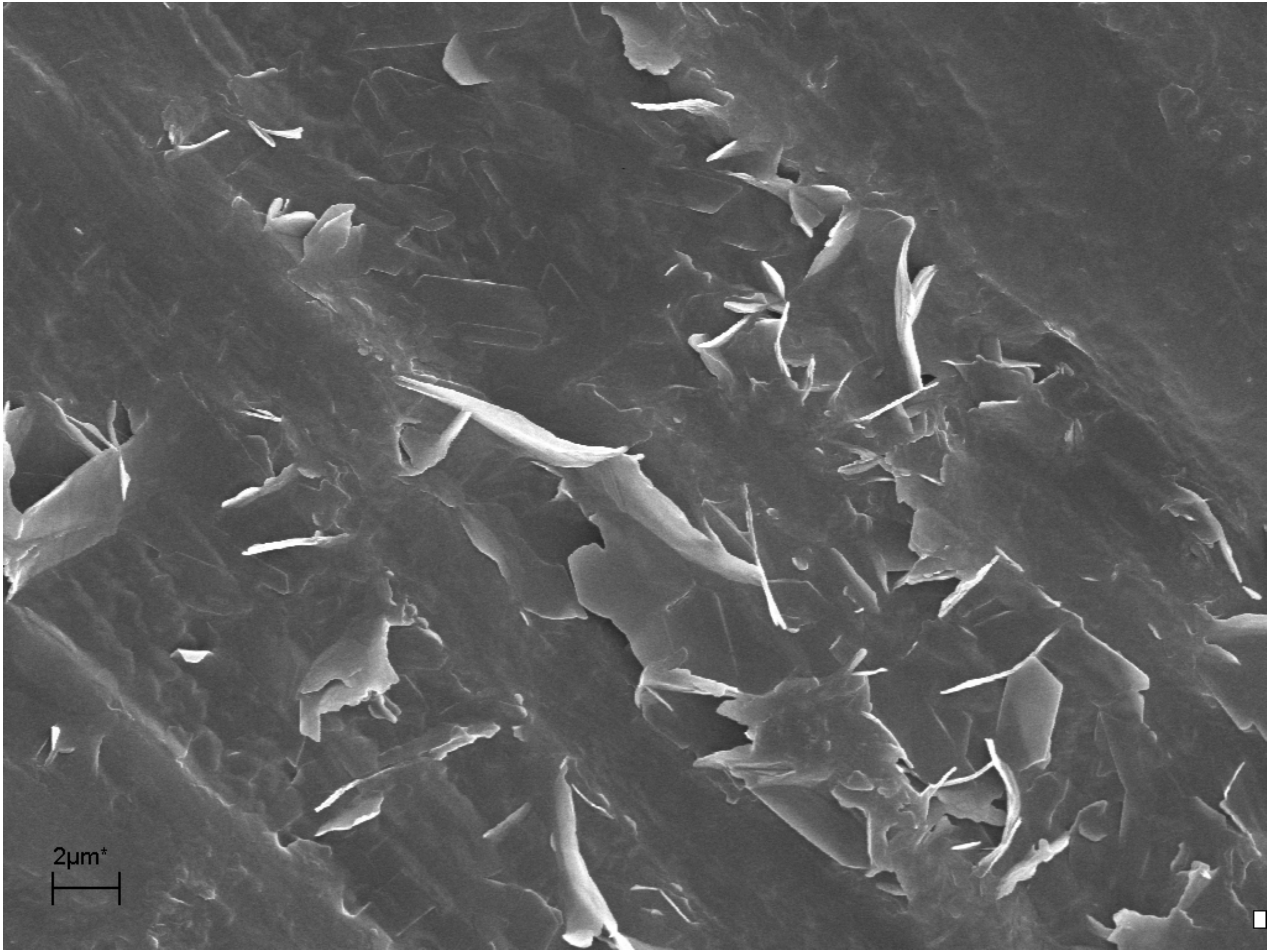

Figure S17. SEM micrographs of AA-CH-S films under 40 kGy gamma radiation dose taken at different magnification

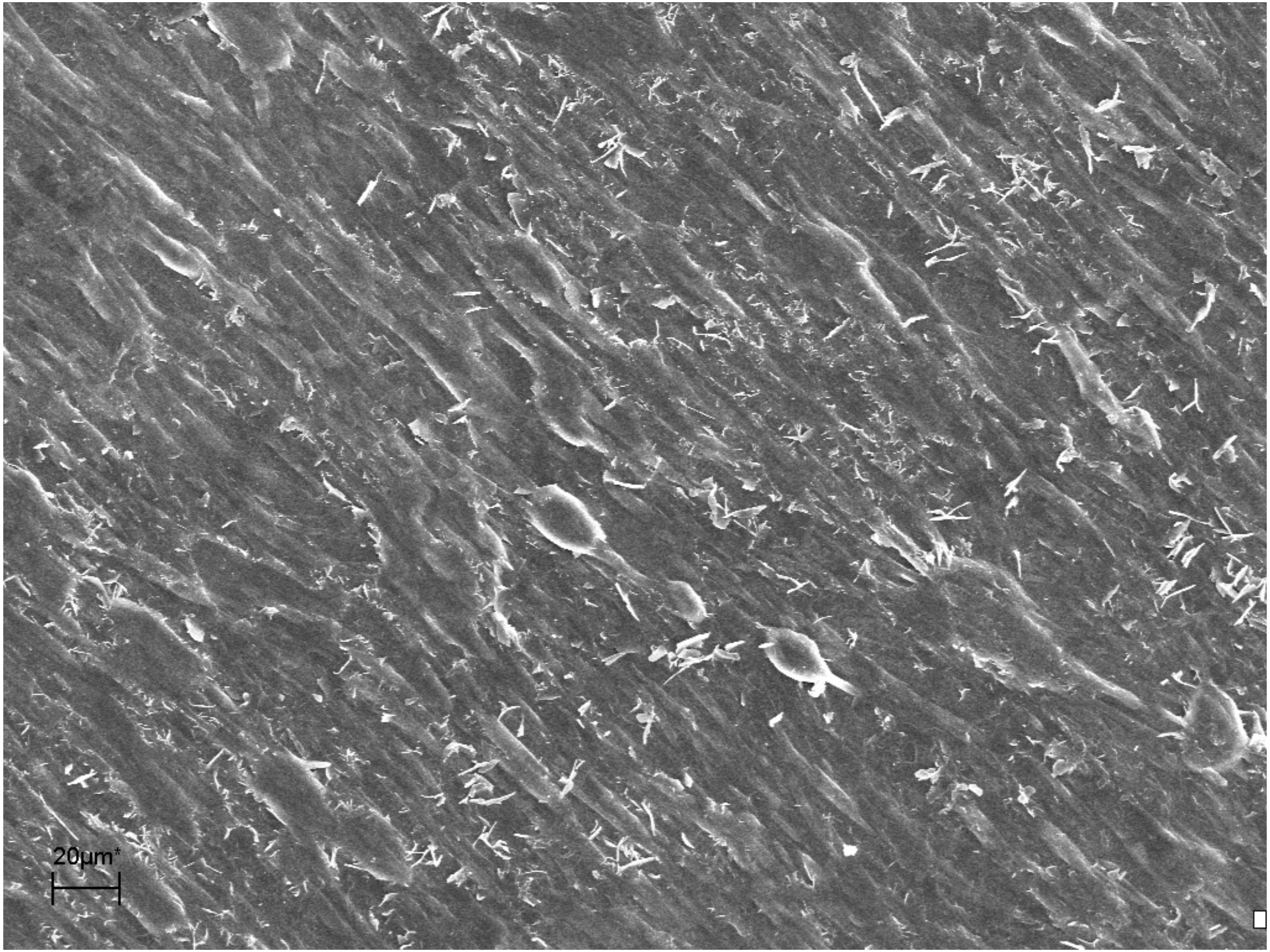

Figure S18. SEM micrographs of AA-CH-S films under 40 kGy gamma radiation dose taken at different magnification

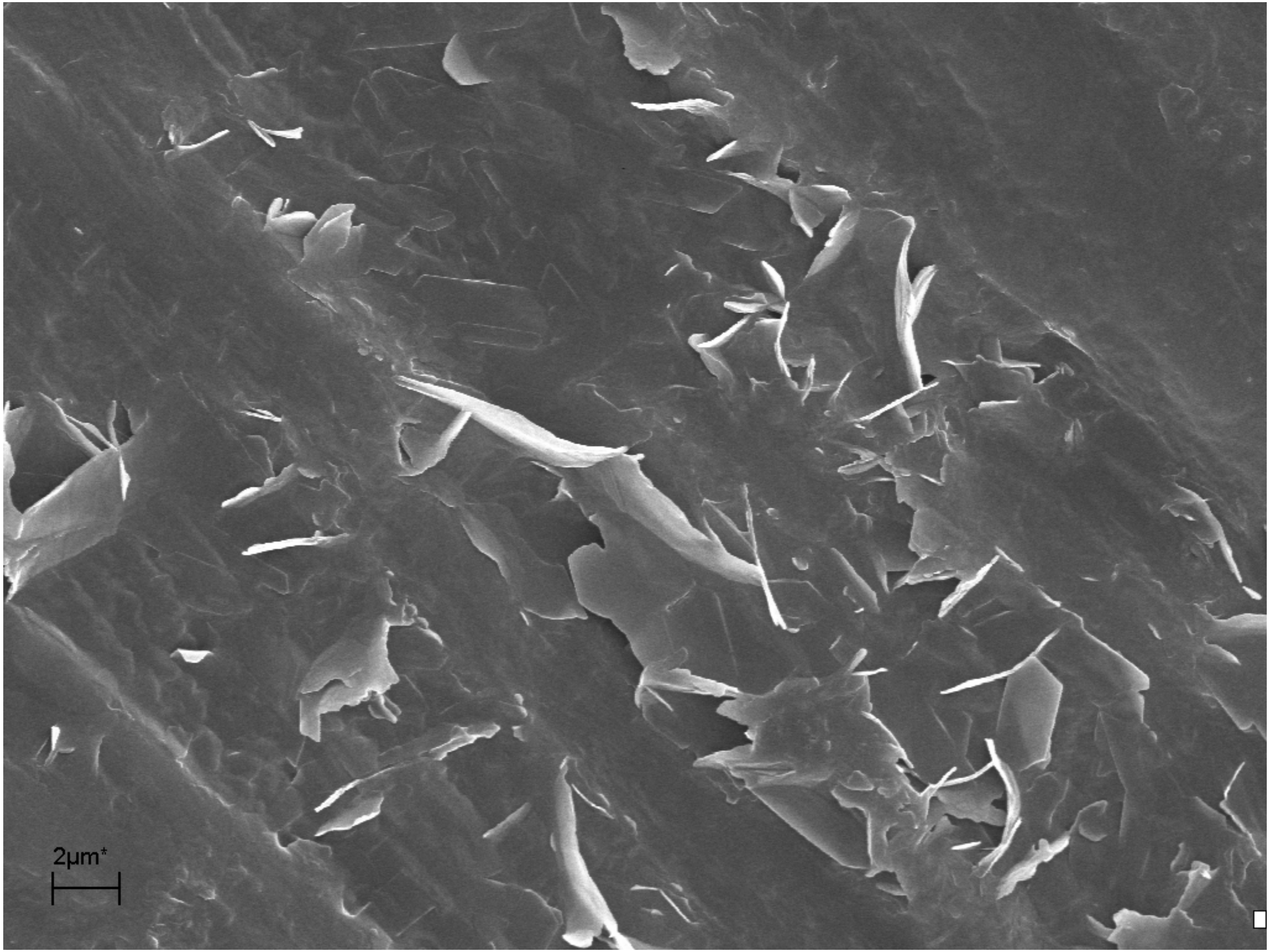

Supplement: Supplementary file 1 [file materials-16-00859-s001.zip › materials-2136668-supplementary/Supplementary Figures S1-S18.pdf]
