# Supplementary material for: Characterization of Biodegradable Food Contact Materials under Gamma-Radiation Treatment
Source: Materials (Basel). 2023 Jan 16;16(2):859. doi: 10.3390/ma16020859 (PMC9861635; doi:10.3390/ma16020859)

Figure S19. SEM micrographs of AA-S films taken at different magnification

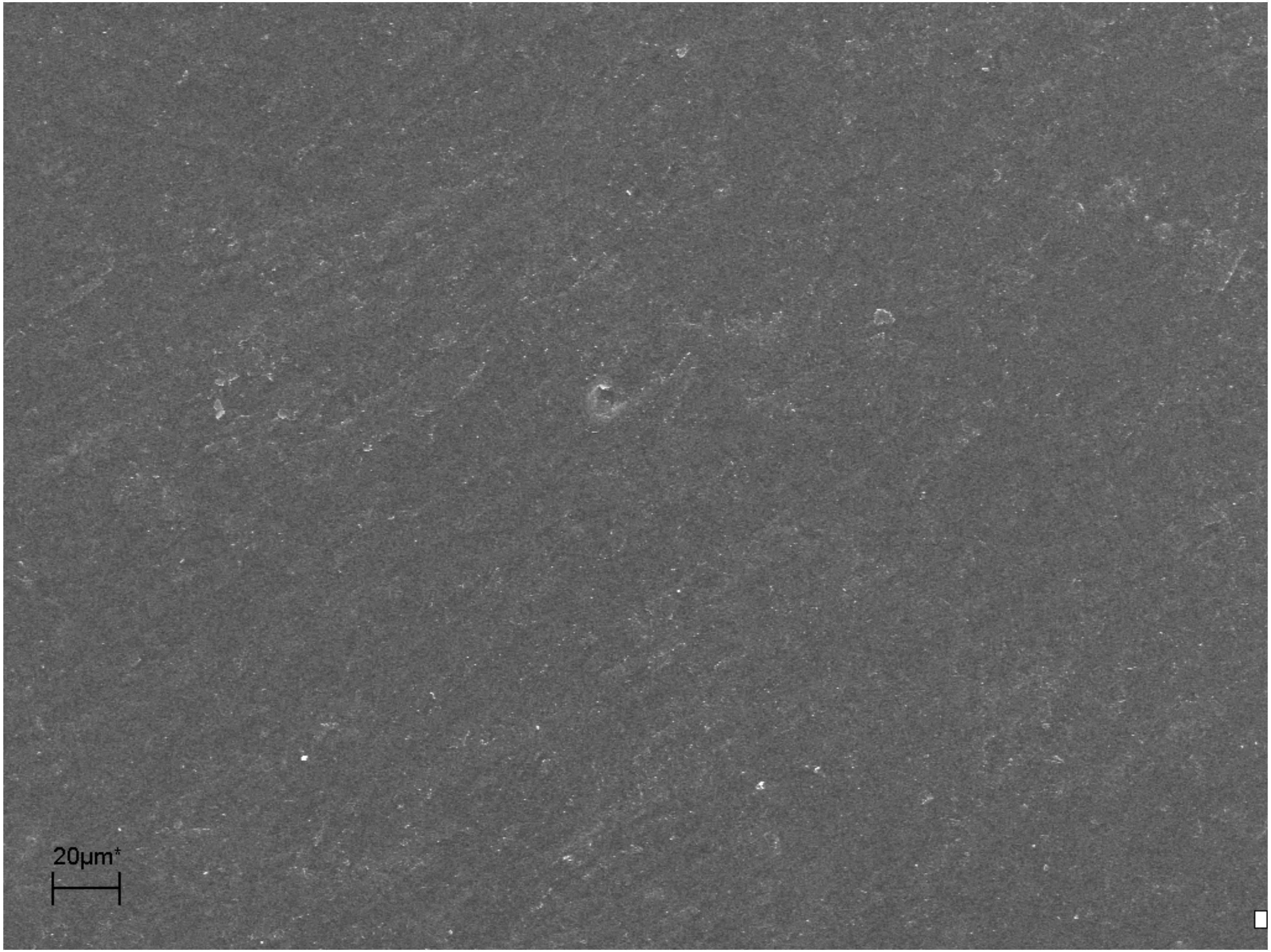

Figure S20. SEM micrographs of AA-S films taken at different magnification

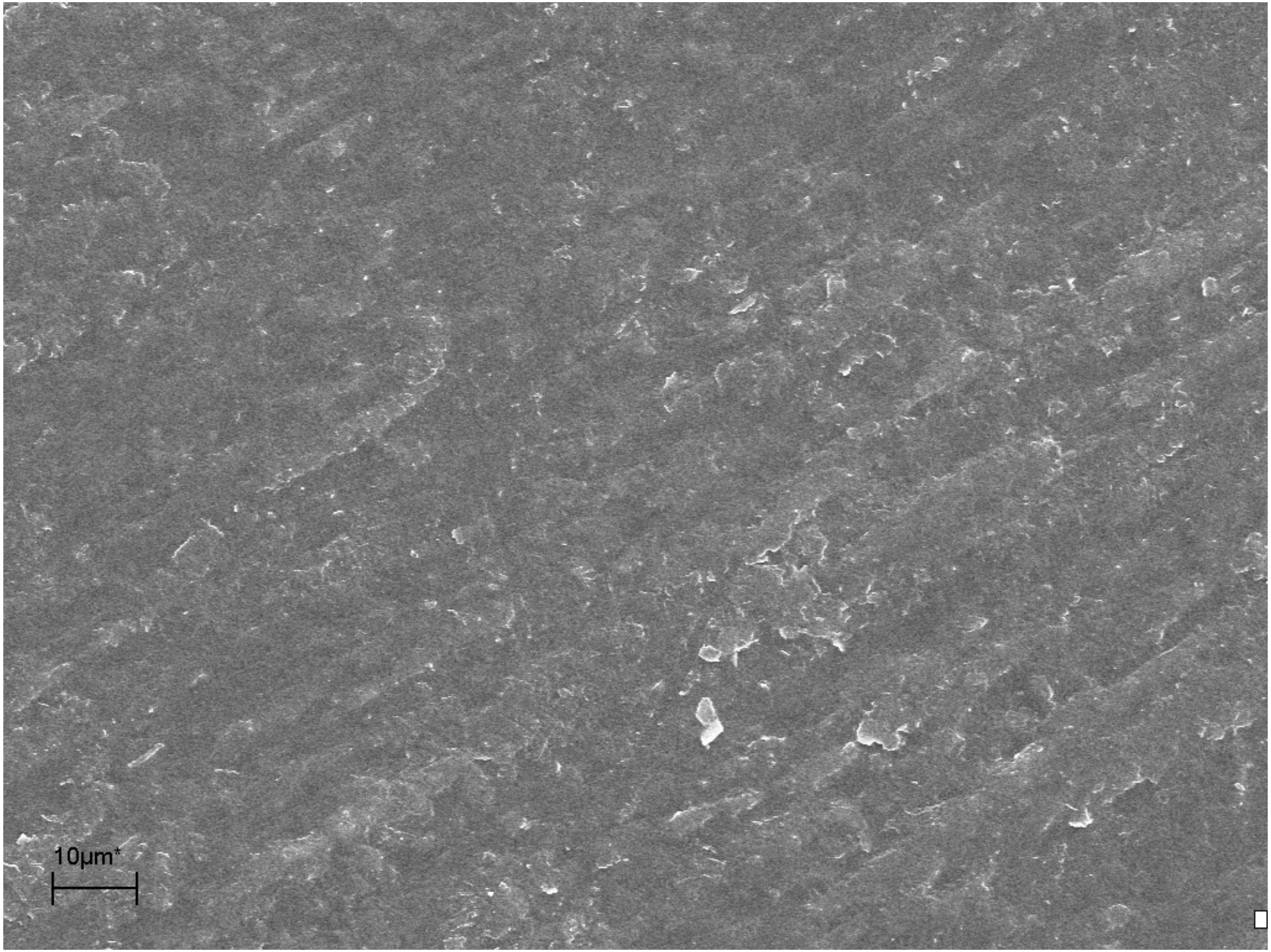

Figure S21. SEM micrographs of AA-S films taken at different magnification

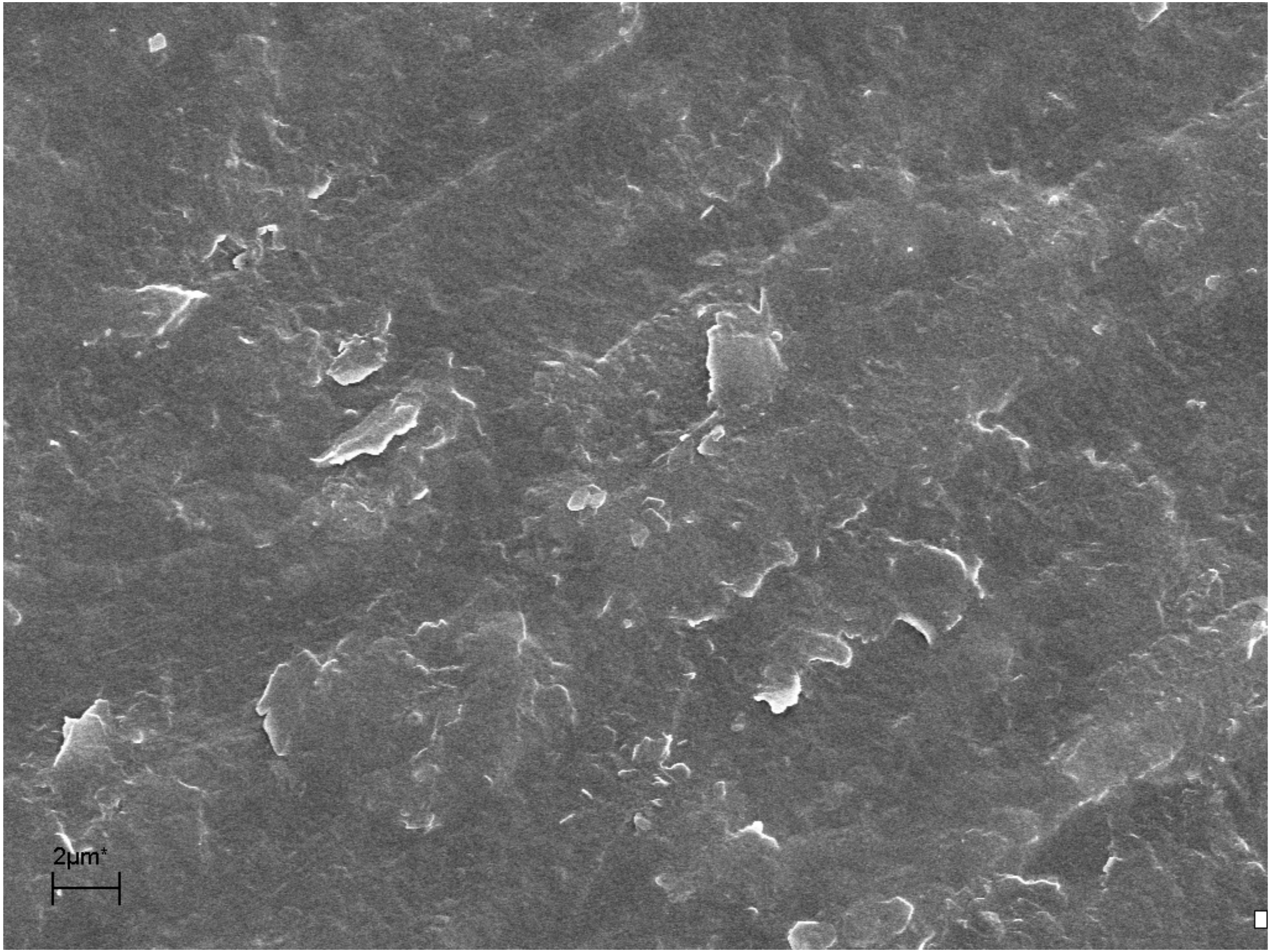

Figure S22. SEM micrographs of AA-S films under 10 kGy gamma radiation dose taken at different magnification

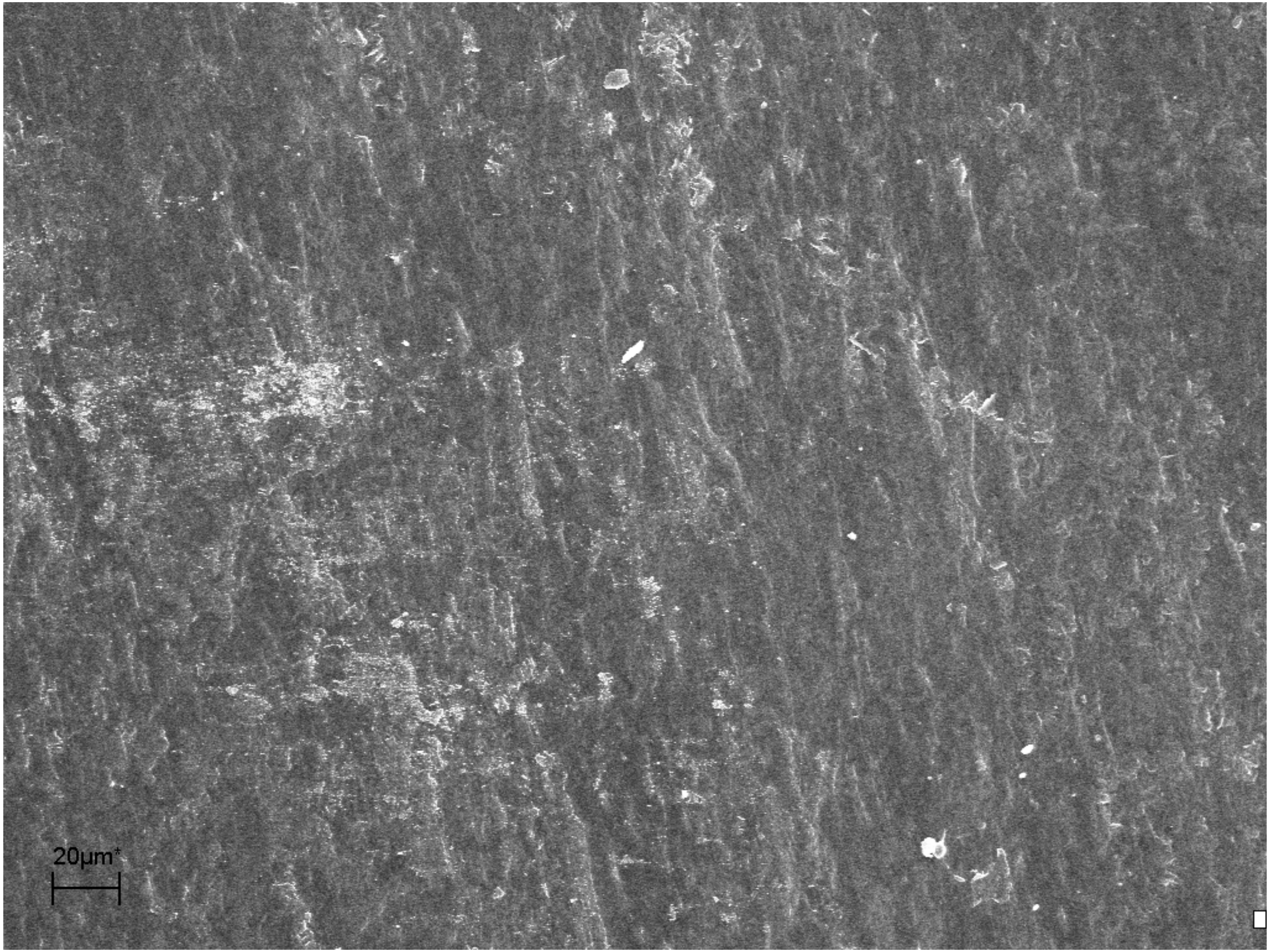

Figure S23. SEM micrographs of AA-S films under 10 kGy gamma radiation dose taken at different magnification

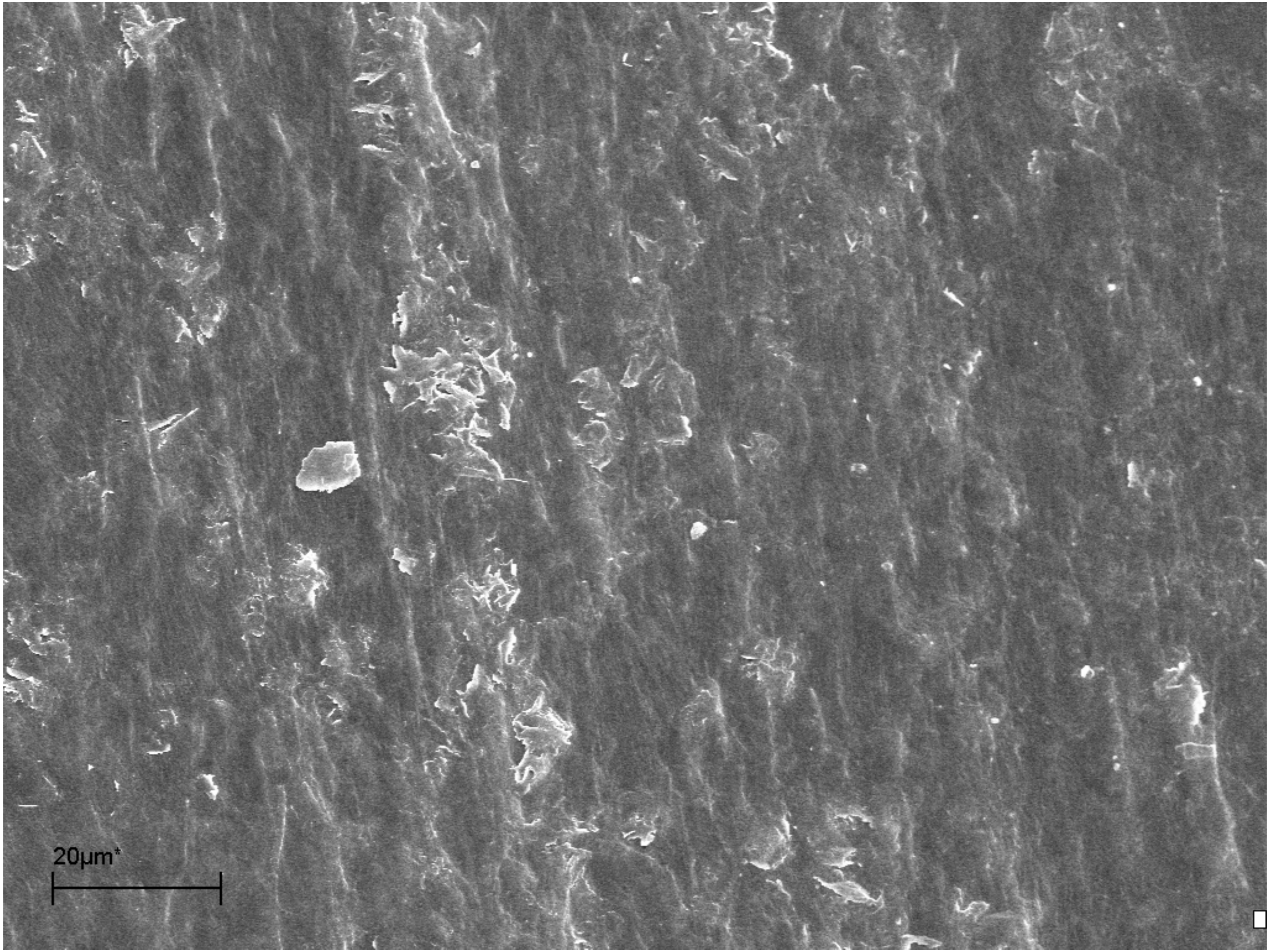

Figure S24. SEM micrographs of AA-S films under 10 kGy gamma radiation dose taken at different magnification

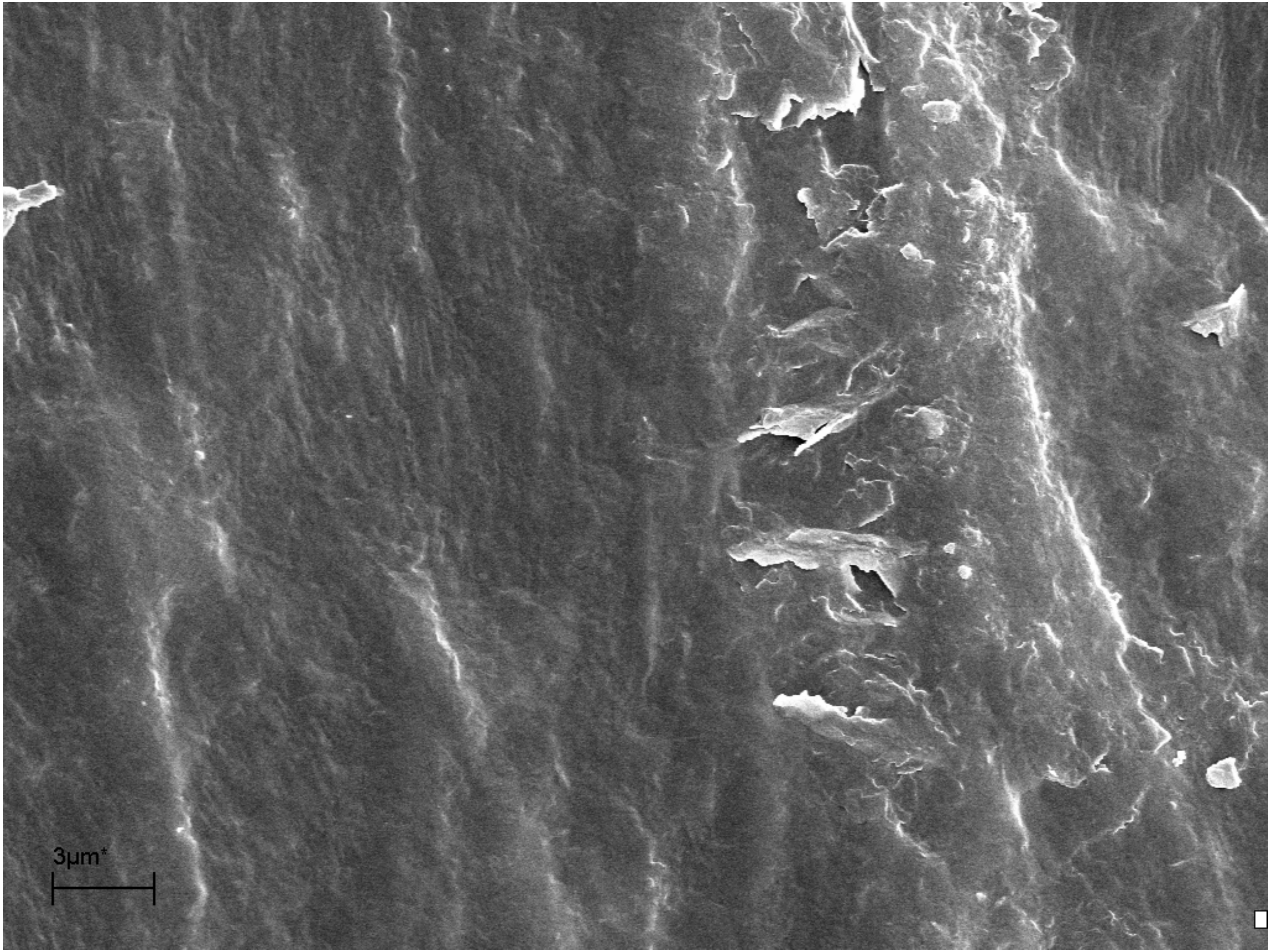

Figure S25. SEM micrographs of AA-S films under 20 kGy gamma radiation dose taken at different magnification

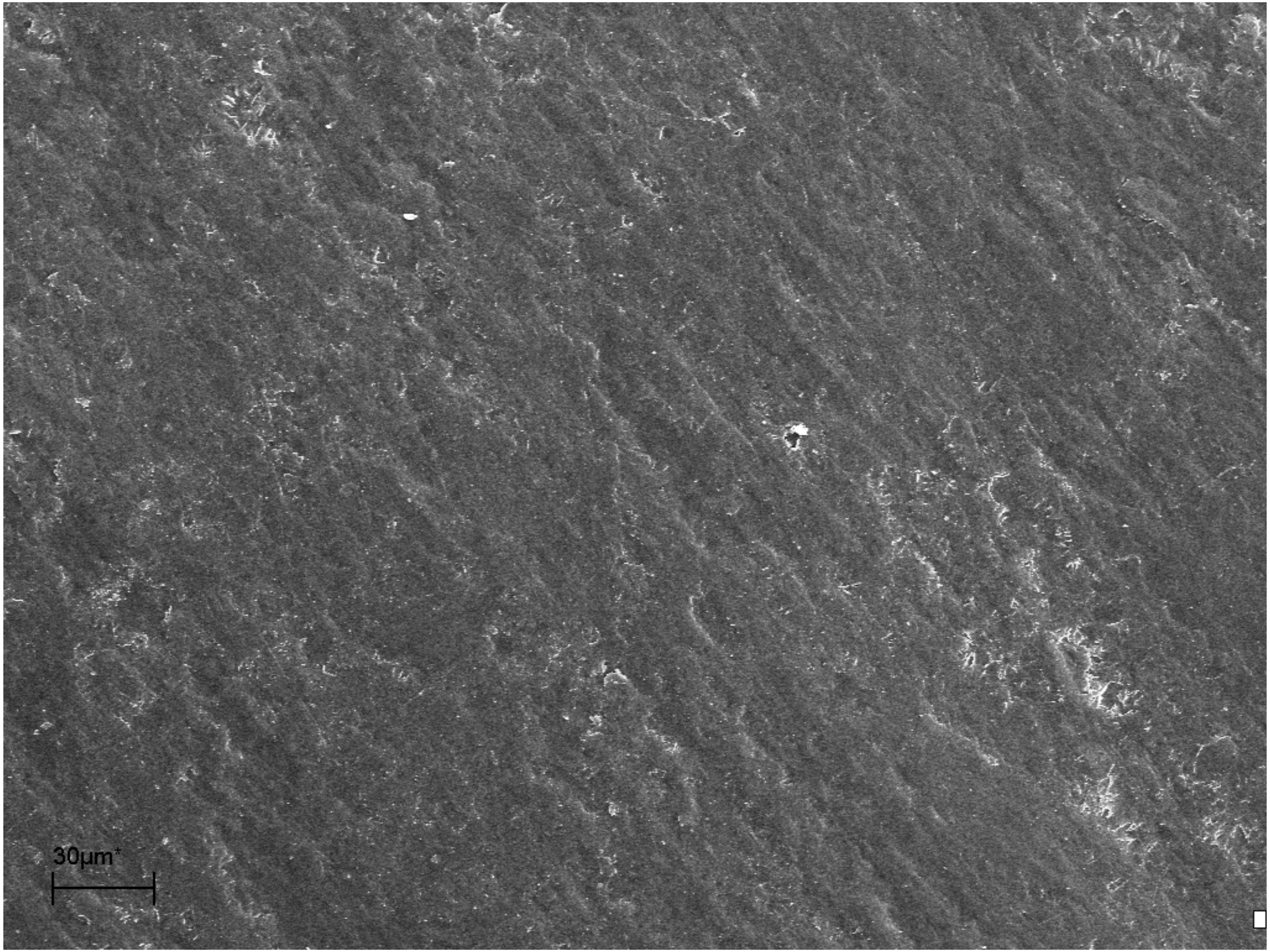

Figure S26. SEM micrographs of AA-S films under 20 kGy gamma radiation dose taken at different magnification

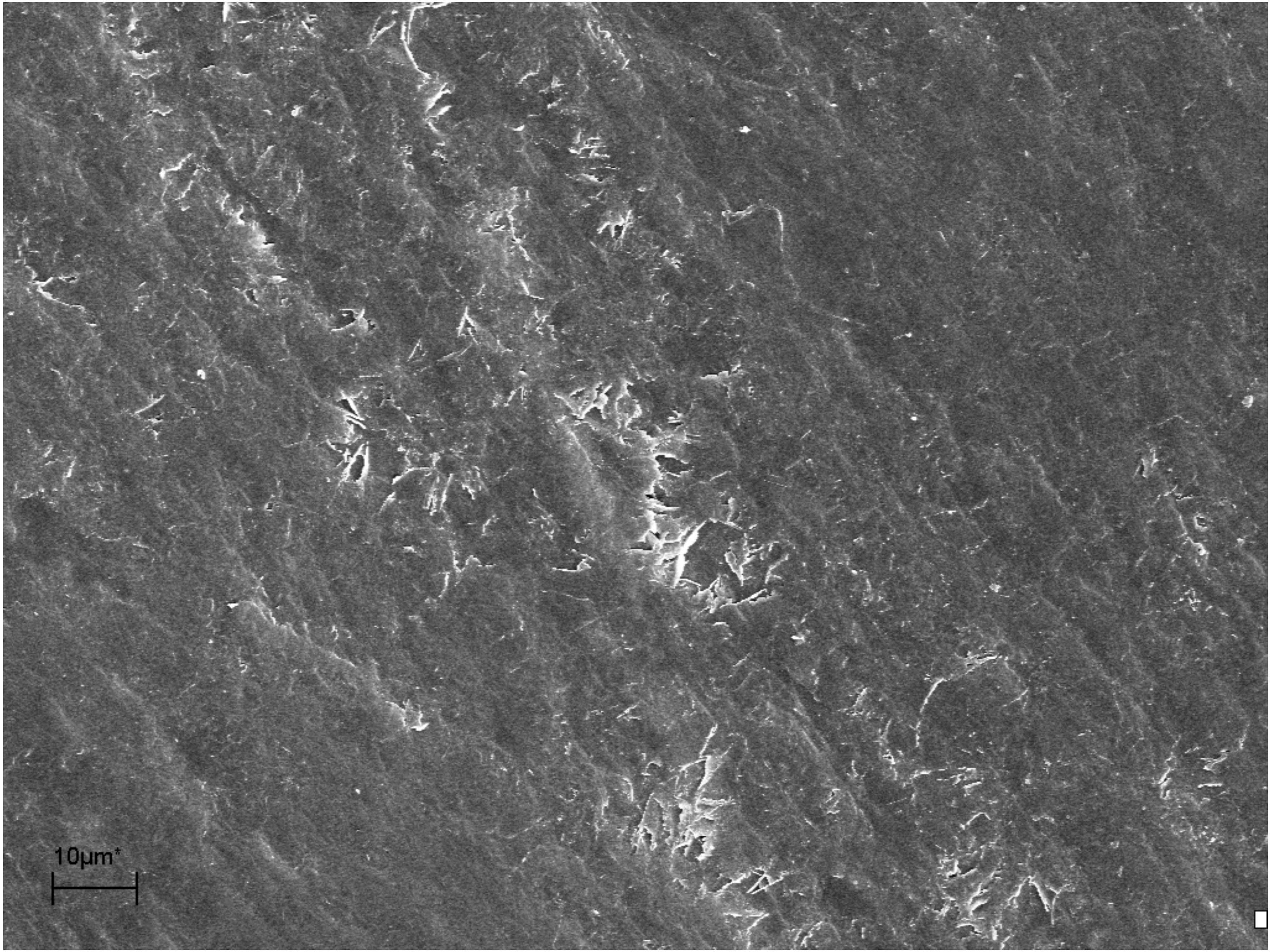

Figure S27. SEM micrographs of AA-S films under 20 kGy gamma radiation dose taken at different magnification

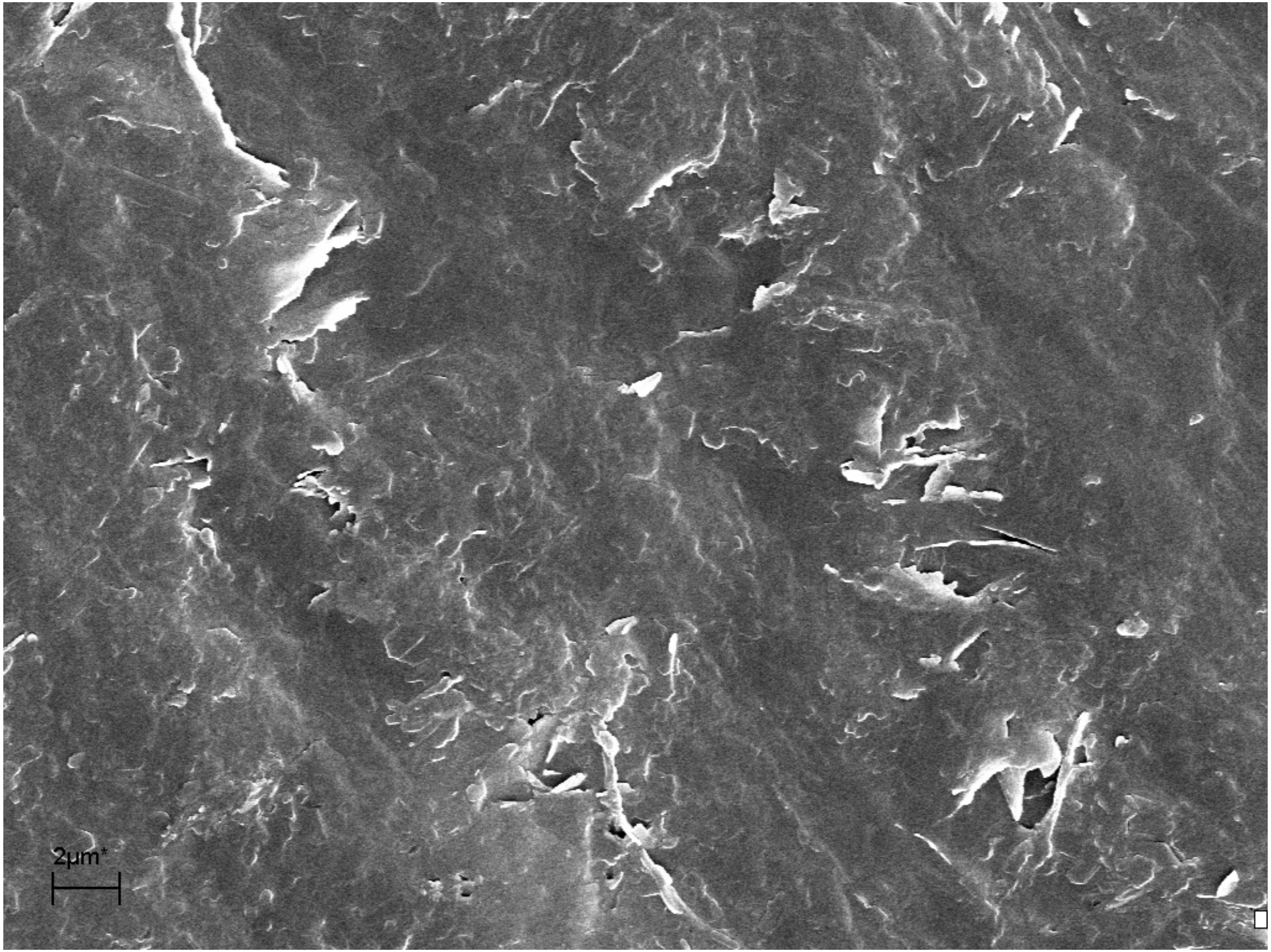

Figure S28. SEM micrographs of AA-S films under 40 kGy gamma radiation dose taken at different magnification

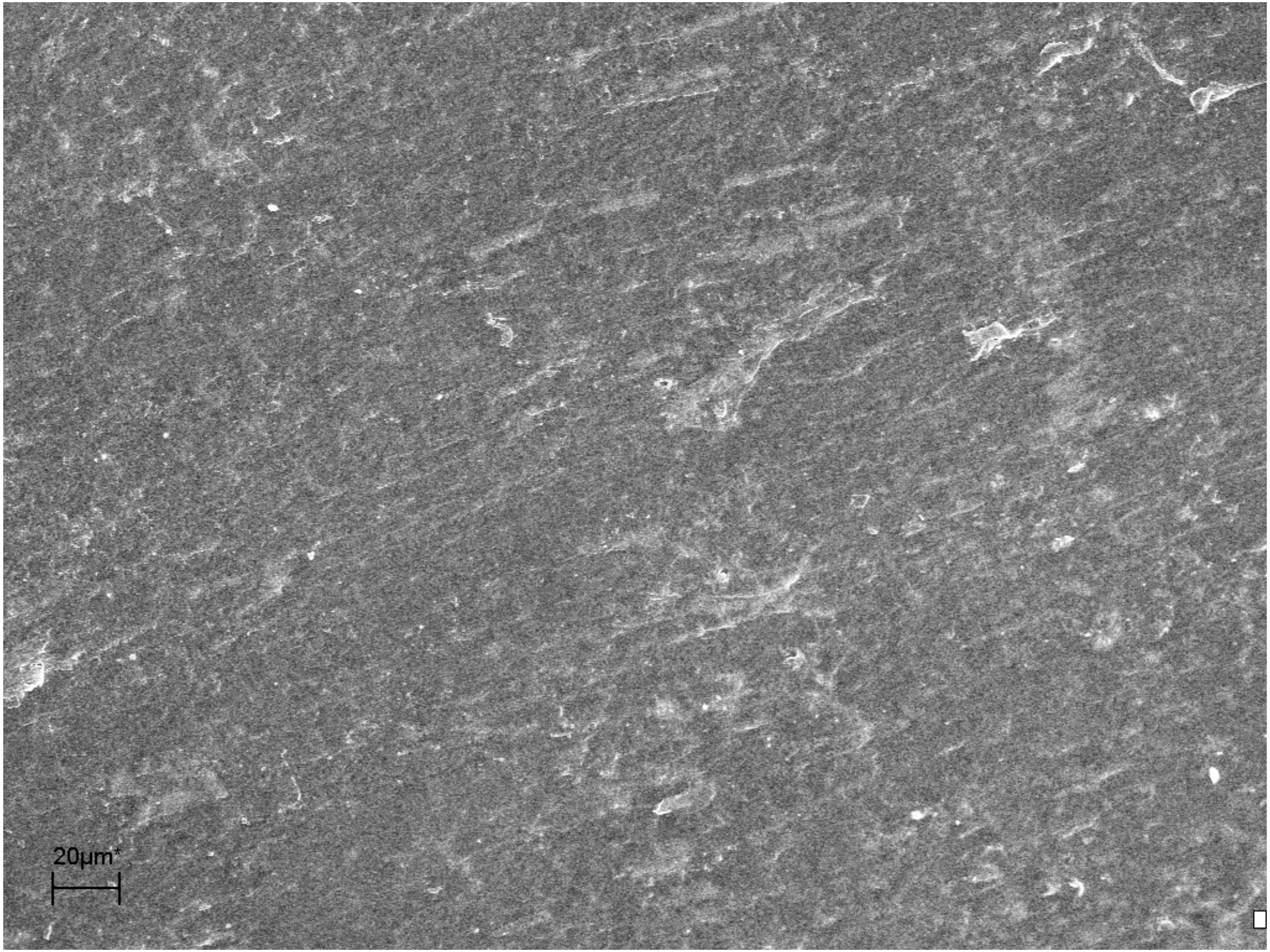

Figure S29. SEM micrographs of AA-S films under 40 kGy gamma radiation dose taken at different magnification

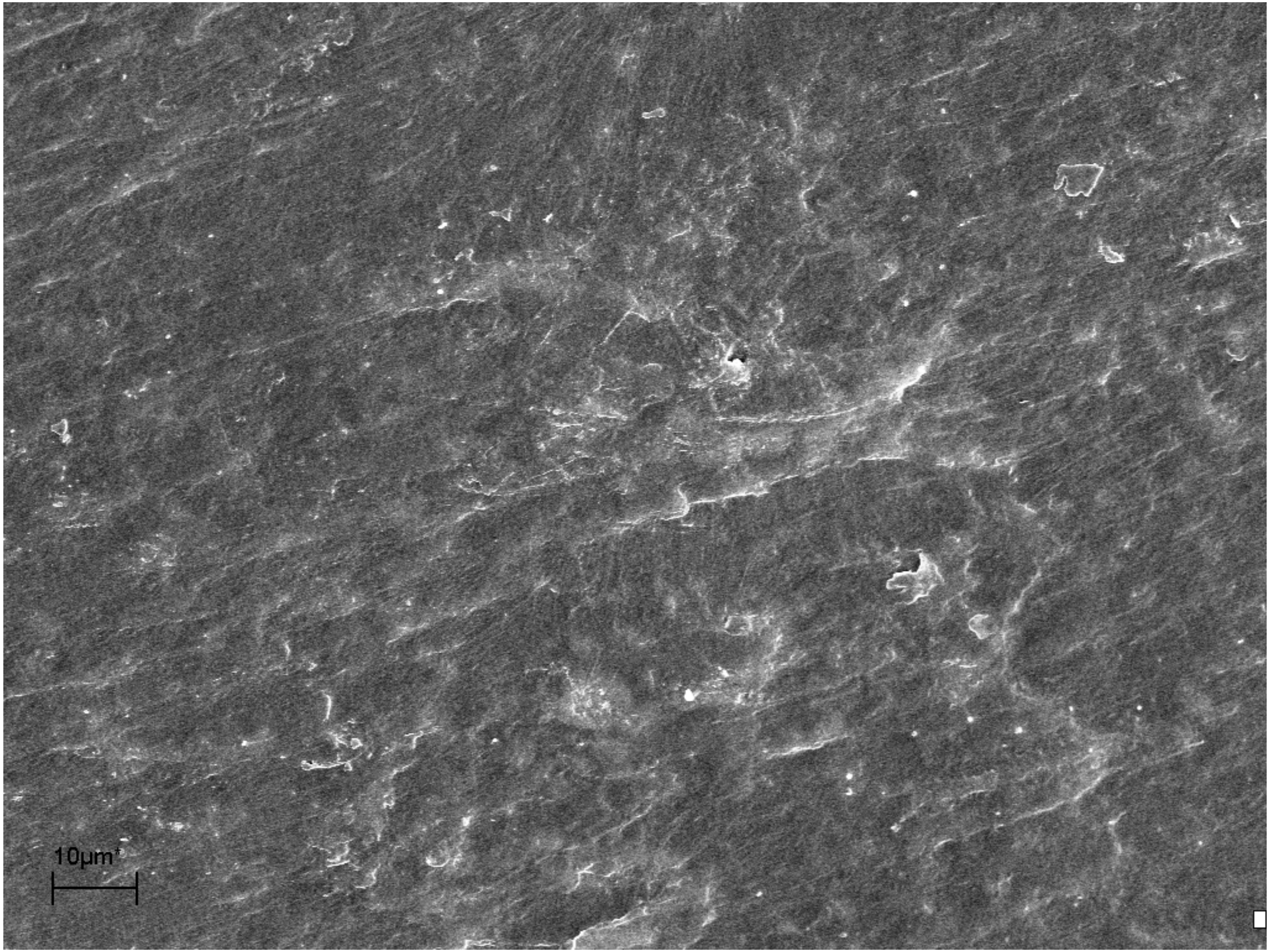

Figure S30. SEM micrographs of AA-S films under 40 kGy gamma radiation dose taken at different magnification

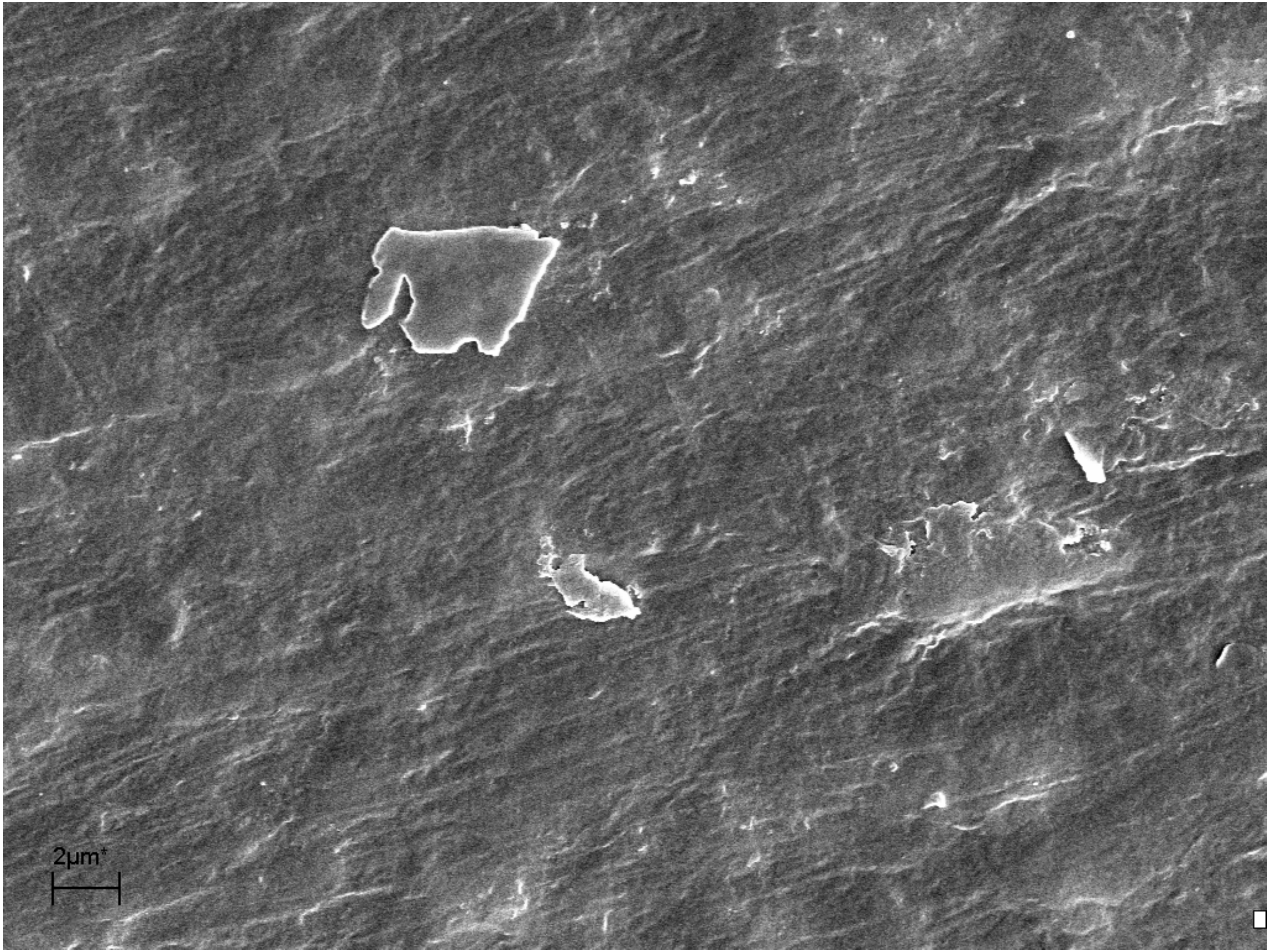

Supplement: Supplementary file 1 [file materials-16-00859-s001.zip › materials-2136668-supplementary/Supplementary Figures S19-S30.pdf]
